# The role of insulin resistance in the development of hepatocellular carcinoma with computational analysis of IRS-1 interaction with HCV genotype 3 core protein

**DOI:** 10.1371/journal.pone.0358858

**Published:** 2026-09-25

**Authors:** Dr Tufael, Mohd Hasan Mujahid, Most Farhana Akter, Tahsin Salam, Md Abu Bakar Siddique, Kaniz Fatima Bari, Asim Debnath, Nabil Deb Nath

**Affiliations:** 1 Department of Biochemistry, Parul University, Vadodara, Gujarat, India; 2 Department of Polymer and Process Engineering, Indian Institute of Technology Roorkee, Uttarakhand, India; 3 Botany Section, School of Sciences, Maulana Azad National Urdu University, Hyderabad, Telangana, India; 4 Department of Pharmacy, University of Development Alternative, Dhaka, Bangladesh; 5 Internal Medicine and Critical Care Medicine, United Medical College Hospital, Dhaka, Bangladesh; 6 Department of Biological Sciences, St John’s University, New York, United States of America; 7 Department of Biochemistry, Shaphena Women’s Dental College, Dhaka, Bangladesh; 8 Burnett School of Biomedical Sciences, College of Medicine, University of Central Florida, Orlando, Florida, United States of America; 9 Department of Biology, The City University of New York, New York, United States of America; Centers for Disease Control and Prevention, UNITED STATES OF AMERICA

## Abstract

The link between insulin resistance (IR) and hepatocellular carcinoma (HCC), especially in relation to HCV genotype-3 infection, is still poorly understood. In South Asian countries and among the peoples of the United States, this problem remains a major public health concern. According to previous studies, metabolic and viral interactomes have not been analyzed together, and hence this is a limitation of the study. This cross-sectional observational study involved 110 people, categorized into control (n=20), chronic hepatitis C (CHC) (n=45), and hepatocellular carcinoma (HCC) (n=45) groups. Clinical, biochemical, and metabolic parameters-HOMA-IR and HbA1c-were measured. The appropriate statistical tests were used for group comparisons. We performed molecular docking to investigate the interaction between the HCV genotype-3 core protein and human IRS-1 using ClusPro. HCC was independently associated with stage III-V fibrosis (AOR: 24.18), elevated HOMA-IR >4, a key indicator of insulin resistance (AOR: 3.53), and AFP >10 ng/mL (AOR: 3.71). Moreover, HbA1c >7% greater GGT and low albumin level were significantly higher in HCC group (P<0.05). Obese patients had higher IR markers, but CHC-to-HCC progression depended more on combined fibrosis and metabolic dysregulation than obesity alone. Docking analysis, there was a strong hydrophobic and electrostatic interaction between HCV core protein and IRS-1, with a binding energy of -1196.0 kcal/mol, indicating a strong and stable interaction between the two proteins. According to this study, it is possible that the presence of insulin resistance, advanced fibrosis, and viral persistence may interact biochemically and molecularly to contribute to hepatocellular carcinoma in cases of HCV genotype-3 infection.

## Introduction

The hepatitis C virus (HCV) constitutes a major global public health concern. The disease’s effects are significantly severe, impacting both developing and developed countries. In South Asia, particularly in Bangladesh, HCV genotype 3 is the most prevalent strain, posing a major regional healthcare challenge. Additionally, it remains significantly represented among immigrant populations in the United States, where its clinical management is also complex and understudied. This genotype is closely associated with hepatic steatosis, metabolic irregularities, and rapid fibrosis progression compared to other genotypes, which can ultimately cause HCC [1–5].

Nonetheless, there is not enough investigation on the influence of insulin resistance (IR) and metabolic variables on liver carcinogenesis linked to HCV genotype-3. Due to this, affected populations, particularly untreated and high-risk individuals in rural and urban areas, are often excluded from early diagnosis and clinical treatment.

Recent findings in the literature show that HCV genotype is associated with viral load, liver injury, and emerging evidence suggests that it is also related to hepatic steatosis and insulin resistance (IR) [6]. While most studies have focused on genotype 1, HCV genotype 3 is increasingly recognized for its distinct metabolic consequences. Compared with other genotypes, genotype 3 has been associated with greater susceptibility to insulin resistance, hepatic steatosis, metabolic disorders, and more rapid fibrosis progression. However, research specifically examining how genotype 3 contributes to metabolic impairment, type 2 diabetes, and progression from chronic hepatitis C to hepatocellular carcinoma (HCC) remains limited. This lack of genotype 3–specific evidence represents an important knowledge gap the present study aims to address [7].

In particular, previous works have not clearly established the collective effects of insulin resistance (IR), fibrosis, and alpha-fetoprotein (AFP) and the direct molecular interaction between the insulin receptor substrate-1 (IRS-1) and the HCV core protein. Most research papers typically only share clinical data; however, there is a notable lack of molecular mechanistic explanation [8,9].

The absence of primary detection of carcinogenic changes in patients infected with HCV genotype 3 is significant and affects early screening, first intervention, and life-saving abilities. In developing countries, where the prevalence of IR and obesity is increasing, such information can be important for the prevention of HCC and making evidence-based policy decisions [10,11].

This study aims to demonstrate the interaction between IRS-1 and HCV core protein. This combination may disrupt the insulin signaling pathway and contribute to the development of hepatocellular carcinoma. Furthermore, integrating fasting insulin, HOMA-IR, HbA1c, fibrosis stage, and AFP offers new insight into the prediction of disease progression and the likelihood of risks [12,13].

The primary objective of this study is to determine the HCC risk in HCV genotype 3 infected patients by examining the relationship between insulin resistance (IR) and liver fibrosis and the tumor marker alpha-fetoprotein (AFP) in these patients [14,15]. This study proposes that HCV genotype 3 interrupts host metabolism and particularly disrupts insulin signaling, which induces insulin resistance, chronic liver damage, and increases HCC risk.

The study is designed to combine clinical observations with in-silico (computational) analyses for this purpose. Experiments for studying the interaction of HCV core protein with host insulin receptor substrate-1 (IRS-1) were carried out using homology modelling and docking studies. How these molecules try to disturb the signaling pathways of insulin is vital to know [16,17]. This research is noted for its mechanistic mixed-method design that integrates clinical and computational approaches.

This study will undergo an analysis using an appropriate statistical method that was conducted to determine the association of Hepatocellular Carcinoma (HCC) with clinical, biochemical, and metabolic variables. The continuous data was analyzed using the Mann-Whitney U test, whereas categorical data was analyzed using chi-square test. To identify the impacts of each risk factor individually, univariate logistic regression was used, whereas to assess the effect of more than one variable at a time, multivariate logistic regression was used [18,19]. By using this statistical framework, the study findings became more trustworthy, and each variable’s independent contribution was easier to identify.

The study presents the complex relationship between insulin resistance (IR) and hepatocellular carcinoma (HCC) in HCV genotype 3 infection through a multidimensional approach combining clinical, biochemical, and in silico analyses. Specifically, the finding that IRS-1 and HCV core protein interact indicates a new biochemical mechanism of hepatocarcinogenesis, the study suggests. Future development of predictive models and therapeutic strategies may be based on these findings, aided by the identification of biomarkers.

## Materials and methods

### Study design and participants

The aim of this study was to ascertain the relationship between insulin resistance and hepatocellular carcinoma (HCC) in patients with HCV genotype 3. This study was conducted based on a cross-sectional observational design and conducted over a period of 16 months, from June 2024 to October 2025, at Ibne Sina Diagnostic & Imaging Center, Dhaka, Bangladesh. The study included 110 patients, categorized into a control group (n = 20), a chronic hepatitis C group (CHC) (n = 45), and a hepatocellular carcinoma group (HCC) (n = 45). Inclusion criteria in this study used the HCV genotype 3 confirmation test. Those who were above 18 years of age and patients of hepatocellular carcinoma (HCC) have to submit evidence from ultrasonic, CT, or MRI reporting, as well as a blood test that would confirm serum collecting alpha-fetoprotein (AFP). The exclusion criteria were co-infection with HBV or HIV, autoimmune hepatitis, alcoholic liver disease, NAFLD, pre-diagnosed type 1 patients, and those taking insulin treatment. The final analysis did not include patients who died throughout the research period.

### Physical examination

At the start of the study, each participant was given a thorough physical examination. The height, weight, blood pressure (BP), peripheral edema, jaundice, splenomegaly, and the presence of ascites were assessed. The size and shape of the liver were checked by palpation. The Body Mass Index (BMI) is calculated by accurately measuring the height and weight. The physical examination was an initial assessment of disease severity and clinical features of cirrhosis, or hepatic decompensation, which was subsequently confirmed by biochemical and radiological assessment.

### Laboratory and biochemical assessments

#### Liver Function Tests (LFTs).

Liver function was determined by using standard biochemical parameters such as ALT (Alanine Aminotransferase), AST (Aspartate Aminotransferase), GGT (Gamma-Glutamyl Transferase), total bilirubin, serum albumin, and INR (International Normalized Ratio) respectively. The tests were performed on an automatic chemistry analyzer like the Vitros 5600 (Ortho Clinical Diagnostics, USA). The INR was done on a dedicated coagulation analyzer the STA Compact Max3 (Diagnostica Stago, France). The following clinical cut-off values were used to define abnormal liver function. They included ALT > 40 U/L, AST > 35 U/L, GGT > 50 U/L for males and >32 U/L for females, total bilirubin >1.2 mg/dL, and serum albumin. 5 g/dL and INR > 1.3 [20,21]. These parameters were used as clinical indicators of hepatic damage, fibrosis, and decompensation.

### Hematological parameter

Platelet count was measured using an automated hematology analyzer the Sysmex XN-1000 (Sysmex Corporation, Japan). Low platelet count (usually below 150 × 10^9^/L) was believed to be an indicator of portal hypertension and cirrhosis [22]. Major drops in platelet levels were considered indirect proof of advanced liver structural damage and fibrosis severity.

### Metabolic parameters

The measures of fasting blood glucose, fasting serum insulin, and HbA1c were done to look at insulin resistance and glycemic control in patients. The HOMA-IR (Homeostatic Model Assessment of Insulin Resistance) was determined based on the following formula as an insulin resistance indicator:


$$\begin{array}{c} HOMA-IR=\frac{\text{Fasting Glucose }(\text{mg}/\text{dL})\times \text{Fasting Insulin }(\mu \text{U}/\text{dL})}{\text{405}} \end{array}$$

The index was utilized in patients to evaluate insulin signaling impairment and the degree of metabolic dysregulation and a HOMA-IR of 2.5 was classified as insulin resistance [23].

### Viral load analysis

Viral loads for HCV RNA are determined by Real-Time Polymerase Chain Reaction (RT-PCR) with the Abbott RealTime HCV assay on the Rotor-Gene Q. The viral load was expressed in IU/mL or as copy numbers. It showed that the virus can replicate and the disease is active.

### Tumor marker analysis

The measurement of serum α-fetoprotein (AFP) levels as a tumor marker for hepatocellular carcinoma (HCC) detection. Use a chemical luminophore immunoassay on the Vitros 5600 (Ortho Clinical Diagnostics, USA) immunoassay machine to quantitate. AFP levels above 20 ng/mL are clinically suggestive of HCC [24], especially considering the patient’s underlying chronic liver disease and insulin resistance.

### Group comparisons and sub-grouping

In order to identify the disease risk factors, the subjects were classified into four groups based on different clinical and metabolic parameters and then studied comparatively:

1. CHC vs. HCC Patient Comparison—A statistical comparison was conducted between CHC and HCC patients to identify high-risk biomarkers among AST, AFP, HbA1c, and HOMA-IR.
2. Obese vs. Non-obese Groups—Patients were classified based on BMI > 25 kg/m². Obesity’s insulin resistance, fibrosis and viral load relationship was analyzed.
3. HOMA-IR > 4 vs. ≤ 4—To determine the relationship between Metabolic Derangement and Liver Injury, patients had divided into Insulin Resistant and Insulin Sensitivity groups.
4. Fibrosis Stage I-II vs. III-V—Patients were grouped in fibrosis stage to check the rate of disease progression and risk of HCC.

### Molecular modeling and docking (*In Silico* analysis)

#### Host and viral protein selection and preterition.

The three-dimensional structure of human insulin receptor substrate-1 (IRS-1) was downloaded directly from the protein databank (PDB ID: 1IRS). The crystal represents the functionally active phosphotyrosine binding (PTB) domain of IRS-1, which plays a crucial role in the first step of the insulin receptor-dependent signaling. The structure was prepared for docking analysis with the free docking software PyRx. The water molecules, cofactors, and nonessential heteroatoms were deleted from the protein with the help of this tool. Furthermore, the structure optimization was done by energy minimization.

The viral core protein was obtained from the amino acid sequence of the HCV core protein genotype-3 from GenBank: EU484153.1. Homology modeling was performed with the Swiss-Model server, using Ornithine carbamoyltransferase (PDB ID: 2ef0.1.A) as the template. The template and the subject were nearly identical with regard to their genetic makeup (99.34% identity and full-length coverage (100%)). We determined the quality of the structural validation GMQE as 0.95, the Global QMEANDisCo score as 0.92 ± 0.05, and the QMEAN Z-score.

### Protein-protein docking and interaction

Analysis of the possible interaction between the core protein of HCV genotype-3 and human IRS-1 was done using the ClusPro 2.0 server. The PDB files that were prepared were uploaded on ClusPro, and rigid-body docking was performed. The server produced possible docking poses of balanced, electrostatic-favored, and hydrophobic-favored. A clustering algorithm was used to group the docked complexes, after which the complex selected from the top-ranked clusters had the lowest binding energy and the highest number of members for interaction interface analysis.

### Ethical approval

The Ethics Committee of Ibne Sina Diagnostic & Imaging Center, Dhaka, Bangladesh, provided ethical approval for this study (Approval No. 11371–5/2024/TAB (715/2024). All procedures were done according to the ethical standards of the 1964 Helsinki Declaration and later amendments [25]. Before enrollment, all participants were provided with written informed consent.

### Sample size and statistical power

A total of 110 participants were included in the study, comprising 20 healthy controls, 45 patients with chronic hepatitis C (CHC), and 45 patients with hepatocellular carcinoma (HCC). The sample size was determined based on pilot data and feasibility, ensuring adequate representation across clinical groups. For sub-group analyses, obese (n = 36) and non-obese (n = 54) participants were compared; although these subgroup sizes are modest, they were considered sufficient to detect clinically meaningful differences in key metabolic and biochemical parameters with an estimated statistical power of 80% at a significance level (α) of 0.05. Group comparisons will be conducted using appropriate statistical tests, including t-tests, Mann–Whitney U test, chi-square, and regression analyses, depending on the variable type and distribution.

### Statistical analysis

The statistical analysis was performed using SPSS (version 25) and R programming. Normality of continuous variables was assessed using the Shapiro–Wilk test. Data were presented as mean ± SD or median (IQR) as appropriate. Based on distribution, ANOVA or Kruskal–Wallis test was used for multiple group comparisons, and independent t-test or Mann–Whitney U test for two-group comparisons. The chi-square test was applied for categorical variables. Univariate and multivariate logistic regression analyses identified predictors of HCC and advanced fibrosis. Odds ratios (OR), adjusted OR (AOR), and 95% CI were reported, with statistical significance set at P < 0.05.

## Results

### Statistical outcome interpretation

#### Demographic characteristics and laboratory parameters analysis.

This research involved the participation of 110 patients, which were assigned to three groups other than the control (n = 20), CHC (n = 45) and HCC (n = 45). While there were age differences in the three groups (control: 34.1 ± 9.7; CHC: 53.2 ± 9.8; HCC: 58.5 ± 9.4 years), they were not statistically significant (P = 0.14). All the groups had similar gender distribution (male: female) (P = 0.84). All groups had similar rates of obesity (control: 40%, CHC: 42%, HCC: 38%; P = 0.69).

In Table 1, the CHC and HCC groups had elevated AST and GGT levels as per the liver function test (AST, control 31, CHC 62, HCC 125 IU/L; GGT, 29, 58, 185 IU/L; P < 0.001). Likewise, bilirubin and INR levels were raised, and albumin levels were decreased in patients with HCC, which means liver function is impaired (Albumin: control 3.9, CHC 3.6, HCC 3.0 g/dL; P < 0.001). Among the biochemical markers, there was a highly significant difference amongst the AFP levels in HCC patients (control: 6, CHC: 32, HCC: 240 ng/mL; P < 0.001). Furthermore, platelet count is significantly lower in the HCC patients (control: 300, HCC: 128 x 10^9^/L; P < 0.001), which is a clinical parameter of cirrhosis.

**Table 1:** Baseline demographics and biochemical profiles of control individuals and patients with chronic hepatitis C (CHC) or hepatocellular carcinoma (HCC).

Variables	Control N = 20	CHC N = 45	HCC N = 45	P-value
Age (Mean ± SD)	34.1 ± 9.7	53.2 ± 9.8	58.5 ± 9.4	0.14
Sex				
Male n = 68	8	27	33	
Female n = 42	12	18	12	
M:F ratio	01:1.5	01:0.67	01:0.36	0.84
Obesity N (%)	8 (40%)	19 (42%)	17 (38%)	0.69
LFT (Median (Range)				
ALT (IU/L)	29 (20-41)	58 (25-205)	65 (34-110)	0.09
AST (IU/L)	31 (18-40)	62 (27-185)	125 (70-310)	<0.001
T.Bil (mg/dL)	0.8 (0.4-1)	1.0 (0.5-3.4)	2.1 (1.1-6.0)	<0.001
Albumin (g/dL)	3.9 (3.5-4.7)	3.6 (1.9-4.3)	3.0 (1.6-3.5)	<0.001
INR	0.8 (0.7-0.9)	1.1 (0.7-1.5)	1.2 (1.1-1.6)	<0.001
GGT (IU/L)	29 (12-46)	58 (15-105)	185 (60-540)	<0.001
Biochemical tests (Median Range)				
Fasting Glu (mg/dL)	99 (80-142)	160 (95-245)	182 (90-880)	0.80
Fasting Ins (μIU/mL)	3.6 (2-5.3)	8.5 (3.5-13.5)	9.5 (4.0-15)	0.17
HbA1c (%)	4.2 (2.7-6)	7.1 (3.3-11.0)	8.2 (4.4-13)	0.05
HOMA-IR	0.87 (0.42-1.49)	3.60 (0.90-7.10)	4.25 (0.95-31.5)	0.64
Plt (×10^9^/L)	300 (152-465)	155 (105-295)	128 (55-180)	<0.001
Cholesterol (mg/dL)	157 (120-247)	205 (160-290)	200 (120-305)	0.95
TG (mg/dL)	153 (125-254)	210 (100-310)	195 (120-310)	0.99
Serum AFP (ng/mL)	6 (2.9-10)	32 (5-120)	240 (150-1080)	<0.001
HCV RNA (viral load copies/IU/L) N (%)				
No viremia	20 (100)			
Low (<100×10^3^)		18 (40)	10 (22)	
Moderate (100-1000×10^3^)		20 (44)	26 (58)	<0.001
High (>1000×10^3^)		7 (16)	9 (20)	
Stage of Fibrosis N (%)				
I + II (n = 20)		14 (31)	6 (13)	<0.001
III + IV + V (n = 70)		31 (69)	39 (87)	

M:F ratio (sex distribution), key liver enzymes (ALT, AST, GGT), total bilirubin (T.Bil), coagulation index (INR), glucose metabolism indicators (fasting glucose, insulin, HbA1c, HOMA-IR), lipid profile (triglycerides), platelet count (Plt), serum tumor marker (AFP), and fibrosis staging (I–II: mild; III–V: advanced) were analyzed.

Analysis of insulin resistance, both fasting insulin and HOMA-IR levels were found to be higher in CHC and HCC as compared to the control (Fasting Insulin: control 3.6, CHC 8.5, HCC 9.5 μIU/mL; HOMA-IR: control 0.87, CHC 3.60, HCC 4.25; not statistically significant P = 0.17, P = 0.64). Nonetheless, the slow accumulation of insulin resistance may imply a modest role in disease progression. In HCC patients, long-term glycated hemoglobin (HbA1c) levels were significantly higher in HCC patients (control: 4.2%, CHC: 7.1%, HCC: 8.2%; P = 0.05).

Analysis of HCV RNA viral load showed that moderate and high levels of viremia were more common in HCC patients (Moderate: CHC 44%, HCC 58%; High: CHC 16%, HCC 20%; P < 0.001). Based on fibrosis stage, a higher proportion of HCC patients were in stages III–V (CHC 69%, HCC 87%; P < 0.001), suggesting that advanced fibrosis may be an important contributing factor in the development of hepatocellular carcinoma.

### Comparison of high-risk variables

A comparative analysis was conducted between CHC and HCC patients for various clinical, biochemical, and viral factors that may contribute to HCC risk, as shown in Table 2. The results demonstrated that a greater proportion of HCC patients were older than 57 years of age (CHC: 40%, HCC: 62%). Furthermore, this was statistically significant (OR: 0.43; 95% CI: 0.19–0.96; P = 0.04). Thus, age may be an independent contributing factor for HCC. The difference OR: 1.33; P = 0.50 was not statistically significant between males and females however, the males (57.1%) were in higher proportion (43.4%). Likewise, obesity (BMI > 25) was roughly the same in both groups (CHC: 42%, HCC: 38%), and no association with HCC risk was seen (P = 0.68).

**Table 2:** Comparative analysis of high-risk clinical variables in HCV Genotype-3 patients with chronic hepatitis C (CHC) versus those with hepatocellular carcinoma (HCC).

Variables	CHC N = 45 n (%)	HCC N = 45 n (%)	Odds ratio (95% CI)	P-value
Age > 57 years	18 (40)	28 (62)	0.43 (0.19–0.96)	0.04
Male gender	27 (60)	30 (67)	1.33 (0.56–3.13)	0.50
Obese (BMI > 25)	19 (42)	17 (38)	1.18 (0.52–2.69)	0.68
LFT				
ALT > 37 IU/L	41 (91)	43 (96)	0.56 (0.10–3.11)	0.72
AST > 40 IU/L	35 (78)	45 (100)	1.33 (1.12–1.59)	< 0.001
T.Bil > 1 mg/dL	22 (49)	45 (100)	2.02 (1.52–2.69)	< 0.001
INR > 1	29 (64)	45 (100)	1.54 (1.25–1.90)	< 0.001
GGT > 50 IU/L	28 (62)	45 (100)	1.68 (1.31–2.12)	< 0.001
Biochemical tests				
Fasting Glucose > 110 mg/dL	42 (93)	40 (89)	1.58 (0.36–6.87)	0.72
Fasting Insulin > 6 μIU/mL	34 (76)	39 (87)	0.49 (0.17–1.41)	0.24
HbA1c > 7%	21 (47)	32 (71)	0.38 (0.16–0.89)	0.02
HOMA-IR > 4	19 (42)	25 (56)	1.67 (0.73–3.79)	0.23
Platelets > 400 × 10^9^/L	0	0	—	—
Cholesterol > 200 mg/dL	18 (40)	19 (42)	1.10 (0.48–2.51)	0.82
TG > 150 mg/dL	43 (96)	43 (96)	1.00 (0.14–6.98)	1.00
Tumor marker				
Serum AFP > 10 ng/mL	30 (67)	44 (98)	25.1 (5.5–112.7)	< 0.001
HCV RNA (viral load)				< 0.001
Moderate (100–1000 × 10^3^)	20 (44)	26 (58)	—	
High (>1000 × 10^3^)	7 (16)	9 (20)	—	
Stage of Fibrosis III–V	31 (69)	39 (87)	22.4 (2.9–81.2)	< 0.001

The clinical variables included body mass index (BMI), liver function tests (ALT, AST, GGT, total bilirubin), coagulation tests (INR; international normalized ratio), metabolic tests (fasting glucose (FG), insulin (FI), HbA1c, insulin resistance (HOMA-IR), and platelet count (Plt)), total cholesterol (TC), triglycerides (TG), and serum AFP. The virological information includes quantification of HCV RNA, viral load (VL), and fibrosis staging, where AF is advanced fibrosis (Stage III–V).

Higher rates of alteration in the liver function markers AST, total bilirubin, INR and GGT were observed in HCC patients, and they all were statistically significant. For example, AST > 40 IU/L was present in 100% of HCC patients and 78% of CHC patients (OR: 1.33; 95% CI: 1.12–1.59; P < 0.001). In the same way, an increase in total bilirubin >1 mg/dL and INR > 1 was present in 100% of the patients with HCC (P < 0.001). In both HCC and CHC patients, elevated GGT levels greater than 50 IU/L were observed. GGT > 50 IU/L was seen in all HCC patients (100%) and 62% of CHC patients. These changes highly indicated the existence of cholestasis or liver damage (OR: 1.68; P < 0.001).

The analysis of biochemical metabolic markers revealed that the proportion of HCC patients with HbA1c > 7% was significantly higher (CHC: 47%, HCC: 71%; OR: 0.38; 95% CI: 0.16–0.89; P = 0.02), which may be attributable to long-term glucose dysregulation and insulin resistance. HCC patients experienced higher rates of fasting insulin >6 μIU/mL and HOMA-IR > 4 but were found to be non-significant (P = 0.24 and P = 0.23) clinically important variables among HCC patients nonetheless.

The presence of serum AFP > 10 ng/mL is a very strong predictor (CHC: 67%, HCC: 98%; OR: 25.1; 95% CI: 5.5–112.7; P < 0.001) and makes it a highly effective tumor biomarker for HCC diagnosis. An examination of the viral load showed that HCC patients had a higher proportion with moderate and high viral loads (≥100 × 10^3^ copies/IU/L) (P < 0.001) but not odds ratio. A noteworthy 69% of the individuals diagnosed with chronic hepatitis C (CHC) and almost all (87%) of the patients with hepatocellular carcinoma (HCC) had stage III-IV fibrosis. Hepatocellular carcinoma was observed to be strongly associated with advanced fibrosis (odds ratio (OR): 22.4; 95% confidence interval (CI): 2.9–81.2; P < 0.001).

### Multivariate analysis of advanced fibrosis factors

A multivariate logistic regression analysis used to assess independent effects of clinical and metabolic factors on development of the HCC study indicates a beneficial polishing effect (Table 3). According to the analysis of the data, those 50 years of age and older were at a greater risk of developing HCC (OR: 2.44; 95% CI: 1.20–4.97; P = 0.012). A body mass index (BMI) more than 25 kg/m^2^ approximately doubled the risk (OR: 1.98; P = 0.024), which shows the role of obesity. Among the liver function markers, higher ALT and AST were associated with 1.8- and 3.06-times higher risk of HCC (P = 0.046 and P = 0.003), and AST was the stronger predictor.

**Table 3:** Multivariate regression analysis identifying independent predictors of advanced fibrosis (Stage ≥ III) in patients with chronic hepatitis C infected with HCV Genotype 3.

Variable	β Coefficient	SE	Odds ratio (95% CI)	P-value
Age > 50 years	0.89	0.36	2.44 (1.20–4.97)	0.012
Male sex	0.45	0.33	1.57 (0.83–2.99)	0.17
BMI > 25 kg/m^2^	0.68	0.29	1.98 (1.12–3.49)	0.024
ALT > 37 IU/L	0.59	0.30	1.80 (1.01–3.24)	0.046
AST > 40 IU/L	1.12	0.38	3.06 (1.44–6.53)	0.003
T.Bil > 1 mg/dL	0.92	0.34	2.51 (1.27–4.94)	0.008
Albumin < 3.5 g/dL	0.83	0.31	2.30 (1.25–4.22)	0.007
INR > 1	0.75	0.28	2.12 (1.19–3.79)	0.011
GGT > 50 IU/L	1.05	0.40	2.86 (1.32–6.20)	0.008
HbA1c > 7%	0.88	0.33	2.41 (1.28–4.55)	0.006
HOMA-IR > 4	1.26	0.42	3.53 (1.54–8.09)	0.003
TG > 150 mg/dL	0.41	0.35	1.50 (0.75–3.01)	0.25
Serum AFP > 10 ng/mL	1.31	0.43	3.71 (1.61–8.52)	0.002
Constant	−2.47	0.58		< 0.001

The multivariate logistic regression models demonstrated acceptable goodness of fit based on the Hosmer–Lemeshow test (P > 0.05). The model performance was further supported by pseudo-R² values, indicating a moderate explanatory power for predicting both advanced fibrosis and hepatocellular carcinoma (HCC). The clinical variables included body mass index (BMI), liver function tests (ALT, AST, GGT, total bilirubin), coagulation tests (INR; international normalized ratio), metabolic tests (fasting glucose (FG), insulin (FI), HbA1c, insulin resistance (HOMA-IR), and platelet count (Plt)), total cholesterol (TC), triglycerides (TG), and serum AFP. The virological information includes quantification of HCV RNA, viral load (VL), and fibrosis staging, where AF is advanced fibrosis (Stage III–V).

Serum bilirubin >1 mg/dL (OR: 2.51; P = 0.008), albumin <3.5 g/dL (OR: 2.30; P = 0.007), and INR > 1 (OR: 2.12; P = 0.011) together clearly indicate liver decompensation and disease progression. Additionally, patients with GGT > 50 IU/L had nearly a threefold higher risk of developing HCC (OR: 2.86; P = 0.008), which may suggest a link to oxidative stress and cholestatic injury.

The presence of HbA1c > 7% among metabolic markers was associated with a 2.4-fold increased risk of HCC (OR: 2.41; P = 0.006). This indicates strong long-term glycemic dysregulation. The odds of HCC development were 3.53 times higher in cases of HOMA-IR > 4 (OR: 3.53; 95% CI: 1.54–8.09; P = 0.003). Insulin signaling disturbance may be directly implicated in HCV genotype 3-related carcinogenesis. Interestingly, serum a-fetoprotein (AFP) >10 ng/mL had the highest increase of HCC risk (OR of 3.71; P = 0.002), making it an effective and clinically useful tumor marker. Being male and having elevated triglycerides, more than 150 mg/dL, didn’t have a significant influence (P value 0.17 and P 0.25). The constant that measured the model fit was statistically highly significant (P < 0.001).

### Multivariate analysis of HCC risk factors

A final logistic regression model was applied to determine the independent associations between clinical and biochemical variables and the risk of hepatocellular carcinoma (HCC) in individuals infected with HCV genotype 3 (Table 4). Results indicate that patients with AST levels greater than 40 IU/L had about six times earlier risk of developing HCC (AOR: 6.12; 95% CI: 1.19–31.44; P = 0.03). Patients with HbA1c > 7% exhibited a noticeable increase in odds (AOR: 2.36), though this was not significant (P = 0.11) as the p-value exceeded the cut-off.

**Table 4:** Multivariate logistic regression identifying predictors of hepatocellular carcinoma (HCC) development in patients with HCV genotype 3 infection.

Factor	P-value	Odds ratio (95% CI)
Age > 57 years	0.63	1.58 (0.55–4.50)
HbA1c > 7%	0.11	2.36 (0.83–6.71)
AST > 40 IU/L	0.03	6.12 (1.19–31.44)
T.Bil > 1 mg/dL	0.28	2.31 (0.41–13.01)
GGT > 50 IU/L	0.21	3.28 (0.56–19.07)
INR > 1	0.39	1.95 (0.46–8.32)
Fibrosis (III–IV)	0.009	24.18 (2.35–281.6)

The multivariate logistic regression models demonstrated acceptable goodness of fit based on the Hosmer–Lemeshow test (P > 0.05). The model performance was further supported by pseudo-R² values, indicating a moderate explanatory power for predicting both advanced fibrosis and hepatocellular carcinoma (HCC). AST (Aspartate aminotransferase), TBil (Total bilirubin), INR (International normalized ratio), GGT (Gamma-glutamyl transferas), HbA1c (Glycated hemoglobin).

Of the other biochemical markers, both GGT > 50 IU/L (AOR: 3.28) and INR > 1 (AOR: 1.95) were associated with increased risk, but their P-values were not statistically significant (0.21 and 0.39 respectively). Likewise, an increase in Total Bilirubin >1 mg/dL resulted in more than two times greater risk (AOR: 2.31), which did not achieve statistical significance (P = 0.28). Patients with Stage III-IV fibrosis had a significantly higher risk for HCC (AOR: 24.18; 95% CI: 2.35–281.6; P = 0.009). This variable was a strong independent predictor in the study.

### Comparison of Obese vs. Non-Obese Patients (CHC & HCC)

In the study, the patients were categorized into two groups based on the body mass index (BMI > 25) obese (n = 36) and non-obese (n = 54). Obese participants (22% of total) had higher fasting plasma glucose (median 182 mg/dL), fasting plasma insulin (10.1 μIU/mL), HbA1c (8.4%), and HOMA-IR (4.9) (p < 0.001 for all), clearly showing evidence of insulin resistance and glycemic dysregulation. This implies that the metabolic instability is more pronounced in obese HCV G3-infected patients (Table 5).

**Table 5:** Comparative analysis of clinical features between obese and non-obese individuals with CHC or HCC infected by HCV genotype 3.

Variables	Obese (n = 36) N (%) / Median (Range)	Non-obese (n = 54) N (%) / Median (Range)	P-value
Age (years)	59 (36–76)	56 (34–73)	0.17
Biochemical tests Median (Range)			
ALT (IU/L)	60 (34–185)	58 (25–110)	0.82
AST (IU/L)	95 (45–310)	85 (27–285)	0.12
T. Bil (mg/dL)	2.0 (0.6–4.5)	1.8 (0.5–5.8)	0.09
Albumin (g/dL)	3.1 (1.7–4.2)	3.3 (1.8–4.3)	0.26
INR	1.2 (0.8–1.5)	1.1 (0.7–1.5)	< 0.001
GGT (IU/L)	82 (30–540)	70 (15–500)	0.043
Fasting Glu (mg/dL)	182 (95–880)	160 (90–870)	< 0.001
Fasting Ins (μIU/mL)	10.1 (6 [–]14 []	7.8 (3.5–15)	< 0.001
HbA1c (%)	8.4 (5.2–12)	6.8 (3.3–13)	< 0.001
HOMA-IR	4.9 (1.7–28.0)	2.5 (0.9–31.5)	< 0.001
Plt (×10^9^/L)	142 (55–250)	138 (60–295)	0.018
Cholesterol (mg/dL)	218 (160–305)	188 (120–250)	< 0.001
TG (mg/dL)	215 (150–310)	185 (100–260)	< 0.001
Tumor marker Median (Range)			
Serum AFP (ng/mL)	120 (6–1080)	175 (5–540)	0.229
HCV RNA (Viral Load IU/L)			
Low (< 100 × 10^3^)	3 (8.3%)	16 (29.6%)	0.001
Moderate (100–1000 × 10^3^)	20 (55.6%)	26 (48.1%)	0.42
High (> 1000 × 10^3^)	13 (36.1%)	12 (22.2%)	0.001
Stage of Fibrosis N (%)			
I + II (n = 20)	2 (10%)	18 (90%)	0.02
III + IV + V (n = 70)	34 (49%)	36 (51%)	

The clinical variables included body mass index (BMI), liver function tests (ALT, AST, GGT, total bilirubin), coagulation tests (INR; international normalized ratio), metabolic tests (fasting glucose (FG), insulin (FI), HbA1c, insulin resistance (HOMA-IR), and platelet count (Plt)), total cholesterol (TC), triglycerides (TG), and serum AFP. The virological information includes quantification of HCV RNA, viral load (VL), and fibrosis staging, where AF is advanced fibrosis (Stage III–V).

Among the liver function markers, the levels of INR and GGT were higher in obese patients, which were recorded at 1.2, 82 IU/L, respectively, and these differences were statistically significant (P < 0.001 and P = 0.043). It could be the result of increased inhibition of liver function or cholestatic damage. Platelet count was slightly more reduced in the obese group (P = 0.018). Nevertheless, other LFT indicators, including ALT, AST, bilirubin, and albumin, showed no significant differences.

The assessment of lipid profile revealed that the obese patients had higher cholesterol (median: 218 mg/dL) and triglyceride (215 mg/dL) levels with a significant difference (both P < 0.001). Non-obese patients had a slightly higher mean level of serum AFP (175 against 120 ng/mL), but this was not a statistically significant value (P = 0.229). In the virologic data, non-obese patients had a higher rate of low viral load (29.6% vs 8.3%; P = 0.001), while the higher rate of high viral load (>1000 × 10^3^ IU/L) was markedly higher in obese patients (36.1% vs 22.2%; P = 0.001).

Most notably, the study explored the occurrence of the fibrosis stage, and the prevalence of Stage I-II was significantly higher among non-obese patients (90% vs. 10%; P = 0.02). This indicates that obese patients are more frequently affected by advanced fibrosis. Stage III–V fibrosis was present in both cohorts. While both arms had a greater number of obese patients (34 vs. 36), this was likely due to insulin resistance and metabolic factors.

### Comparison between CHC and HCC patients with HOMA-IR

The comparison of CHC and HCC groups in patients with insulin resistance (HOMA-IR > 4) was made with the statistical treatment. Although both groups recorded approximately the same proportion of patients aged over 57 and male (63–64% and 68%, respectively), important differences were seen in other clinical and biochemical parameters. The prevalence of obesity (BMI > 25 kg/m^2^) in CHC patients was higher (79%) than that in HCC patients (64%). Insulin resistance probably plays a role in the development of HCC. However, obesity alone may not be the case for HCC (Table 6).

**Table 6:** Comparison between CHC and HCC patients with HOMA-IR > 4 HCV Genotype 3.

Variables	CHC (HOMA-IR > 4) n = 19 (42%)	HCC (HOMA-IR > 4) n = 25 (56%)
Age > 57 years	12 (63%)	16 (64%)
Male gender	13 (68%)	17 (68%)
Obese (BMI > 25 kg/m²)	15 (79%)	16 (64%)
LFT (Median ± Range)		
ALT > 37 IU/L	17 (89%)	23 (92%)
AST > 40 IU/L	17 (89%)	25 (100%)
T. Bil > 1 mg/dL	13 (68%)	25 (100%)
INR > 1	17 (89%)	25 (100%)
GGT > 50 IU/L	16 (84%)	25 (100%)
Biochemical tests		
Fasting Glucose > 110 mg/dL	19 (100%)	25 (100%)
Fasting Insulin > 6 μIU/mL	19 (100%)	25 (100%)
HbA1c > 7%	18 (95%)	24 (96%)
Cholesterol > 200 mg/dL	13 (68%)	14 (56%)
TG > 150 mg/dL	19 (100%)	24 (96%)
Tumor marker		
Serum AFP > 10 ng/mL	19 (100%)	25 (100%)
HCV RNA (viral load)		
Moderate (100–1000 × 10^3^)	11 (58%)	18 (72%)
High (> 1000 × 10^3^)	7 (37%)	7 (28%)
Stage of Fibrosis		
III + IV + V	18 (95%)	25 (100%)

BMI (Body mass index), LFTs (Liver function tests), ALT (Alanine aminotransferase), AST (Aspartate aminotransferase), TBil (Total bilirubin), INR (International normalized ratio), GGT (Gamma-glutamyl transferas), FG (Fasting glucose), FI (Fasting insulin), HbA1c (Glycated hemoglobin), HOMA-IR (Homeostasis model assessment of insulin resistance), Plt (Platelet count), TC (Total cholesterol), TG (Triglycerides), AFP (Alpha-fetoprotein), HCV RNA (Hepatitis C virus ribonucleic acid), VL (Viral load), AF -Advanced fibrosis (Stages III–V).

In the HCC group, all patients had liver function indicators of AST > 40 IU/L, bilirubin >1 mg/dL, INR > 1, and GGT > 50 IU/L. The CHC group also had raised levels of these markers but they were not present in all patients (AST: 89%, bilirubin: 68%, INR: 89%, GGT: 84%). Metabolic dysregulation was clear in all the study subjects in both groups, as fasting glucose was > 110 mg/dL with fasting insulin >6 μIU/mL and HOMA-IR > 4. Moreover, the percentages of HbA1c > 7% were also very high (CHC: 95%, HCC: 96%), indicating a long-standing effect of insulin resistance. The rate of triglyceride >150 mg/dL was found to be 100% in the CHC group and 96% in HCC. However, relatively higher cholesterol (>200 mg/dL) was observed in CHC patients (68% vs. 56%).

Both groups (100%) had serum AFP > 10 ng/mL. This tumor biomarker is already active in patients with insulin resistance. These two groups are at an increased risk of hepatocarcinogenesis. In HCC patients, moderate viral load (100–1000 × 10^3^ IU/L) was more frequent (72% vs. 58%). However, the proportion of high viral load (>1000 × 10^3^ IU/L) patients was slightly higher in CHC patients (37% vs. 28%). These findings could hint at a complicated link between viral activity and insulin resistance. Both groups were classified at fibrosis stage III-V (CHC: 95%, HCC: 100%). This shows that with HOMA-IR > 4, the majority of patients were already at an advanced fibrosis stage. This may give a valuable basis for progression to HCC.

### Association of HOMA-IR with liver fibrosis and viral load

**Fig 1:**
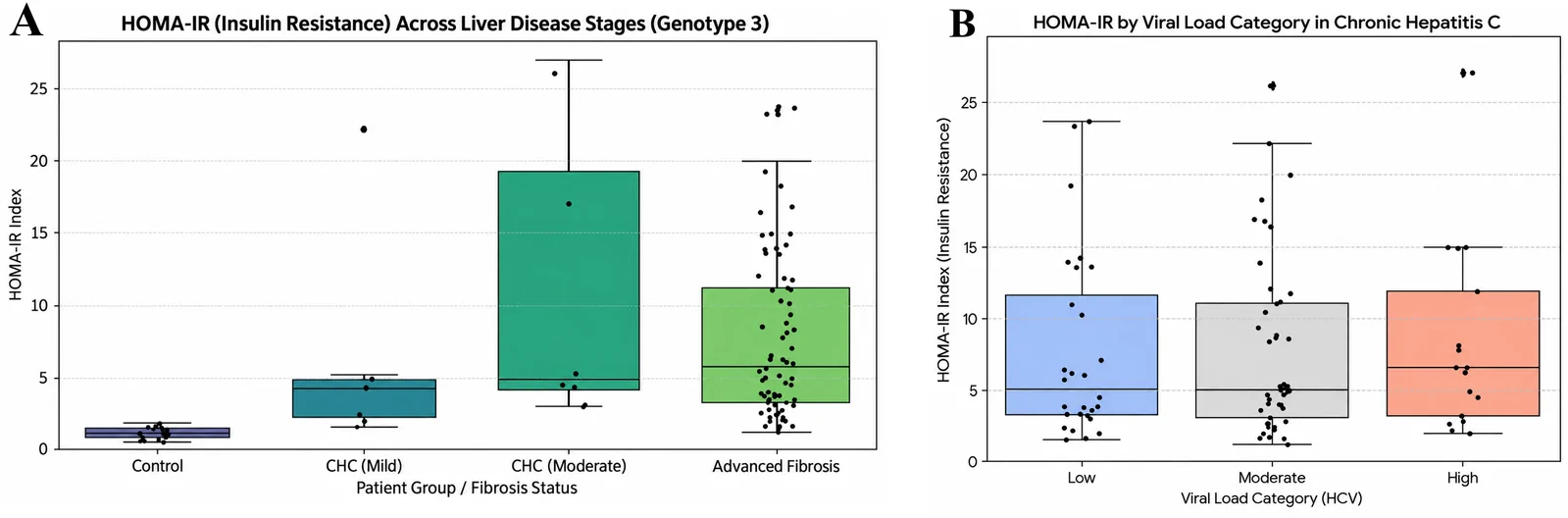
(A) HOMA-IR progressively increases across liver disease stages, with the lowest values in healthy controls, a moderate elevation in CHC (moderate fibrosis), and the highest levels in advanced fibrosis. This trend highlights that insulin resistance worsens as liver fibrosis progresses in genotype-3 HCV infection. (B) Higher HOMA-IR variability is observed in patients with high HCV viral load compared with low or moderate viral load. These trends together suggest that both fibrosis progression and higher viral persistence contribute to elevated insulin resistance in genotype-3 HCV-infected individuals.

The progressive nature of liver disease can be shown by the increasing insulin resistance (HOMA-IR index) as depicted in Fig 1A. The HOMA-IR is close to 1 in the control group, shows moderate elevation in CHC patients with mild or moderate fibrosis, and peaks in patients with advanced fibrosis, with several values exceeding 15–20. This indicates that insulin resistance worsens as liver fibrosis progresses, suggesting a cause-and-effect relationship between metabolic dysregulation and liver damage, and supporting its potential role as a disease-promoting factor.

The HOMA-IR levels also vary with HCV viral load, as shown in Fig 1B. While low, moderate, and high viral load groups partially overlap, patients with high viral load exhibit greater variability and some HOMA-IR scores above 15–20. Together, these trends suggest that in HCV genotype-3 infection, viral persistence may contribute not only to liver damage but also to metabolic alterations.

### *In-Silico* analysis

#### Pathway mapping analysis.

**Fig 2:**
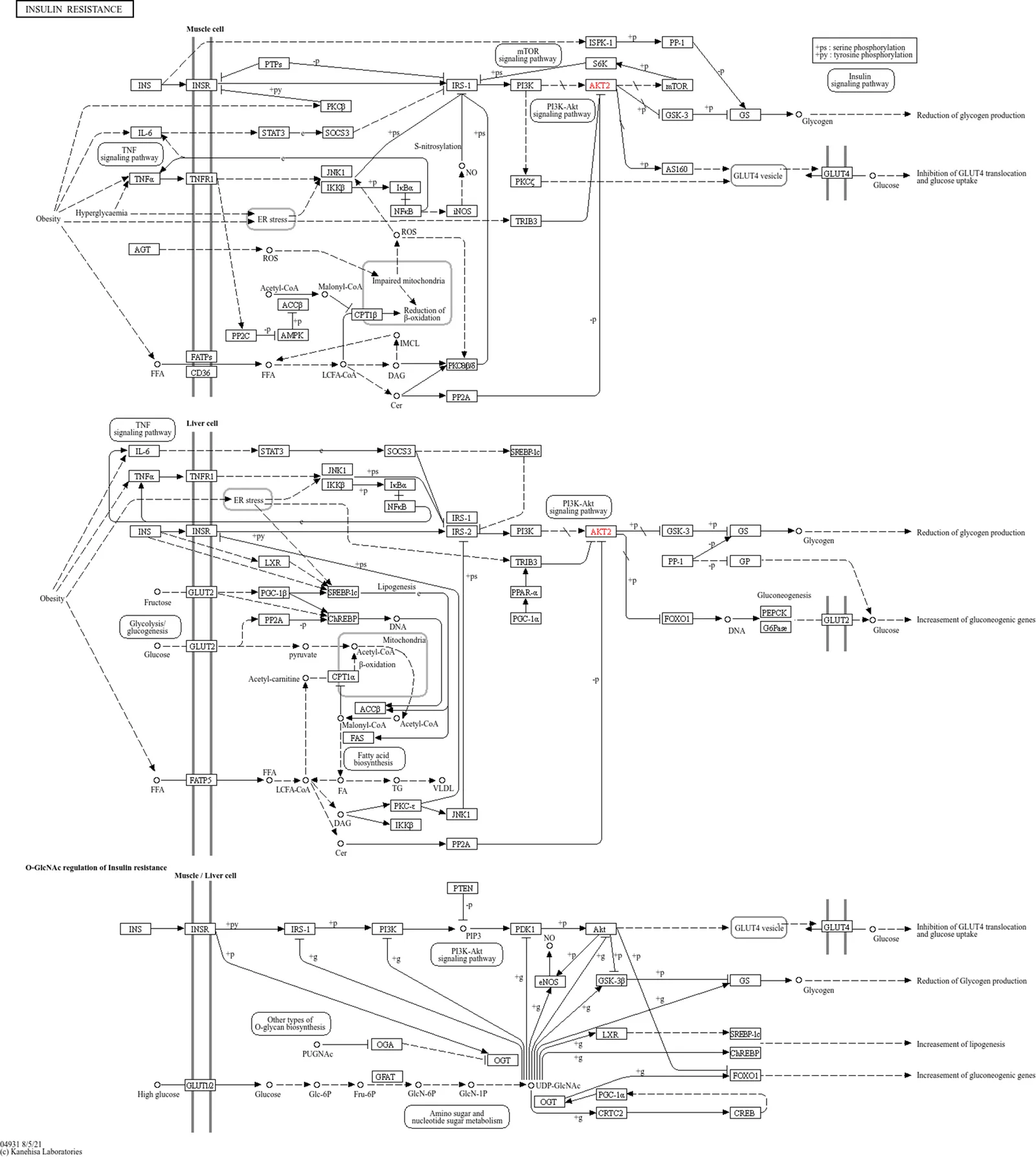
The KEGG insulin resistance pathway (hsa04931), showing the predicted interference of HCV Core Protein with IRS-1. The pink-highlighted proteins, including IRS-1, AKT, GLUT4, mTOR, and FoxO1, are primarily involved in the viral-induced disruption, leading to altered glucose homeostasis, hepatic insulin resistance, and acting as risk factor for hepatocarcinogenesis.

In Fig 2 illustrates the KEGG insulin resistance pathway (hsa04931). IRS-1 and AKT are key nodes of insulin signal transduction. The in-silico docking analysis shows that HCV genotype-3 core protein binds IRS-1, disrupting the PI3K/AKT cascade. This interference impairs GLUT4 translocation, glycogen synthesis, and inhibits gluconeogenesis, leading to hepatic insulin resistance. The pink-highlighted proteins (IRS-1, AKT, GLUT4, mTOR, and FoxO1) are the most affected by this interaction, and their disruption may increase the risk of hepatocellular carcinoma (HCC). These trends summarize the impact of HCV core protein interaction on insulin signaling as depicted in the pathway diagram**.**

### Homology modeling analysis

**Fig 3:**
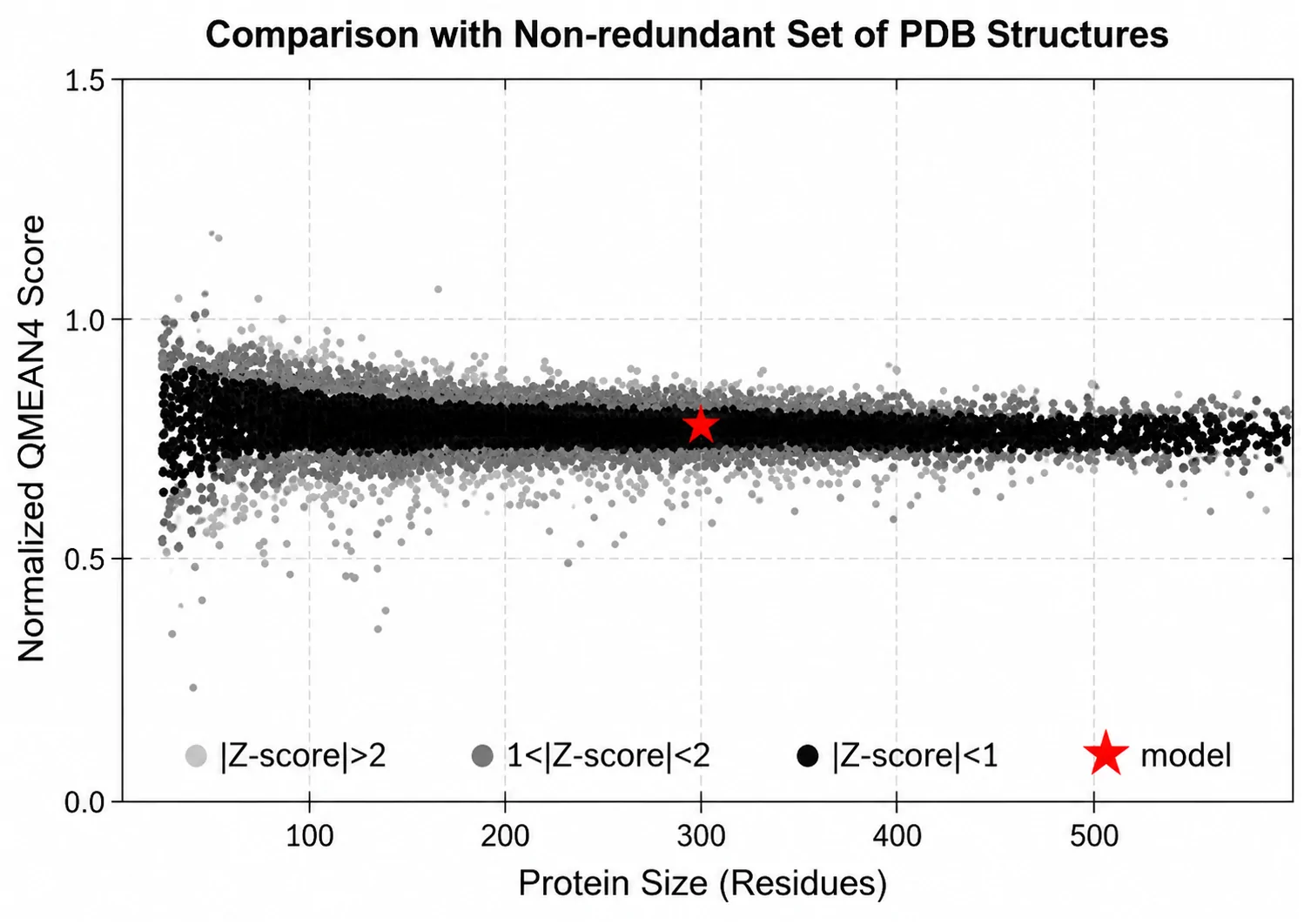
Structural validation for homology-modeled HCV Core Protein from QMEAN Z-score plot. The red star shows the position of the model relative to a non-redundant set of experimentally derived PDB structures. The Z-score is within the acceptable limit or range (|Z-score| < 1), suggesting a good match with high-quality structural templates.

Using the Swiss-Model Server, the three-dimensional structure of HCV Core Protein was modelled for structural analysis of protein-protein interaction with IRS-1. The modeling was done using ornithine carbamoyltransferase (PDB ID: 2ef0.1.A) as the template. The chosen template has a very high sequence identity with the target protein (99.34%) and ensures full-length coverage (~100%). The quality of the structure is high with a resolution of 2.0 Å. The high GMQE score of the present model (0.95) and QMEANDisCo Global Score of 0.92 ± 0.05 indicate good reliability and structural robustness (Fig 3).

Analysis of local quality estimation indicated most of the residues displayed a predicted local similarity score greater than 0.9. The region between residue numbers 210−240 has scores that are lower (0.4–0.6). These are more likely part of flexible loops or disordered regions. Solvent-exposed and interaction-prone surfaces may influence docking interactions in these areas.

The model fits well with other PDB non-redundant protein structures as per the QMEAN Z-score visualization. In other words, a Z-score of | < 1| indicates acceptance of the protein model’s structure. The ligand binding analysis indicated that the target model is in a ligand-free conformation, despite the presence of MAGNESIUM and SODIUM ions in the template, none of them were in direct contact with the target model.

### Molecular docking and interaction analysis

**Fig 4:**
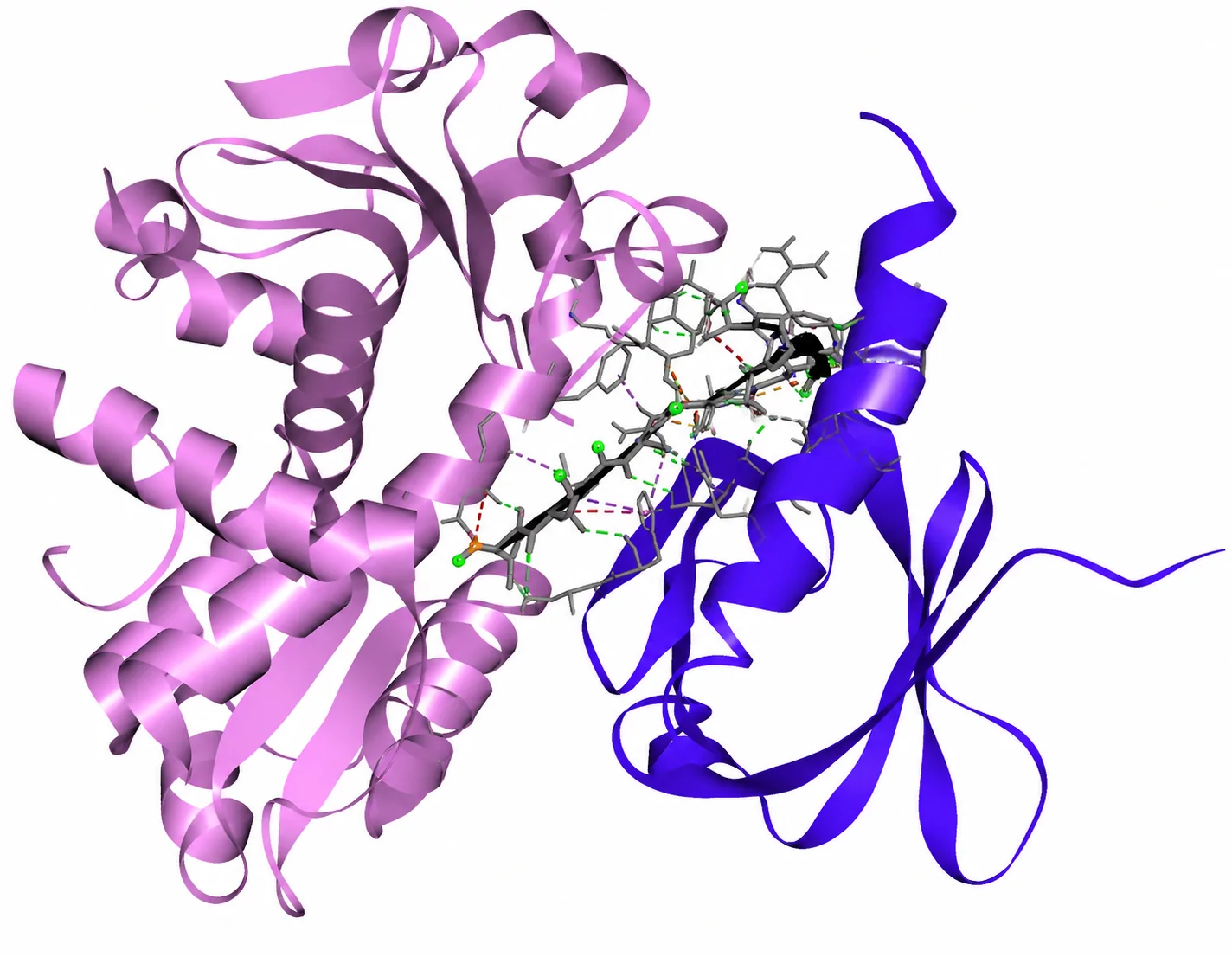
Protein-protein docking clusters between HCV core protein (blue) and IRS-1 (pink). Cluster 0 exhibited the most stable complex, (−1196.0 Kcal/mol) suggesting a strong presence of hydrophobic interaction.

To understand the protein-protein interaction, docking analysis between the HCV Core Protein and IRS-1 was performed using the ZDOCK and ClusPro servers. Two types of configurations-hydrophobic-favored and electrostatic-favored were used in the docking process to better comprehend the interaction dynamics (Fig 4).

The hydrophobic interaction-favored docking produced the largest cluster of 128 models, which is cluster 0. Notably, the weighted score of the cluster’s central configuration was −1103.4 kcal/mol with a minimum energy score of −1196.0 kcal/mol. The energy scores of Clusters 1 (−1115.4 kcal/mol), Cluster 3 (−1145.7 kcal/mol), and Cluster 9 (−1139.6 kcal/mol) also show significant low energy. The binding configurations of these clusters often revealed overlapping interfaces involved in binding to the HCV Core protein that included Arg117, Leu139, and Phe132 that interacted with Asp378, Tyr465, and Glu445 of IRS-1.

Clustering of the docked complexes revealed that Clusters 2, 3, 5, and 6 had the lowest weighted energy scores (−878.0 kcal/mol, −918.3 kcal/mol, −965.8 kcal/mol, and −962.3 kcal/mol, respectively) for the docking favored with electrostatic interaction. It is observed that Cluster 5 (−965.8 kcal/mol) represents the most stable interaction, as it has the lowest energy score among the four clusters discussed. The energy scores have shown a slight increase relative to the ones observed in the hydrophobic-favored docking. Nevertheless, some clusters exhibited a reasonable intermolecular attraction as well as favorable electrostatic binding.

The docking modes reveal that while the HCV Core Protein makes primary hydrophobic contacts with IRS-1, the presence of electrostatic interactions confers additional stability, which assists with the complex formation. The binding pockets found in the best-ranked clusters are solvent accessible and located near the functionally relevant PTB domain. The interfaces predicted to bind are in the 1200–1300 Å^2^ range. Such ranges are common in protein-protein complexes.

### Binding affinity analysis

Protein-protein interaction analysis done by ClusPro proves a total of 18 clusters for the docking configuration favored by electrostatics shown in Table 7. Different illustrative structures invoked by each cluster may represent one of the potential complex HCV Core Protein (blue) and IRS-1 (pink). Cluster 5 has the lowest weighted energy score (−965.8 kcal/mol), making it the most stable in electrostatic interaction. Clusters 6 (−962.3 kcal/mol), 8 (−917.9 kcal/mol), and 3 (−918.3 kcal/mol) also exhibit very low energy scores, indicating that there could be several strong electrostatic binding modes between IRS-1 and HCV Core Protein.

**Table 7:** Electrostatic docking clusters with energy scores and stability assessment.

Cluster	Members	Representative	Weighted Score (kcal/mol)
0	81	Center	−848.7
		Lowest Energy	−848.7
1	69	Center	−852.4
		Lowest Energy	−958
2	64	Center	−868.2
		Lowest Energy	−878
3	59	Center	−870.2
		Lowest Energy	−918.3
4	54	Center	−845.9
		Lowest Energy	−906.5
5	52	Center	−811.3
		Lowest Energy	−965.8
6	46	Center	−790.6
		Lowest Energy	−962.3
7	44	Center	−759.1
		Lowest Energy	−833.3
8	42	Center	−763.5
		Lowest Energy	−917.9
9	34	Center	−746.7
		Lowest Energy	−888.4
10	29	Center	−786.8
		Lowest Energy	−838.4
11	25	Center	−762.5
		Lowest Energy	−850.2
12	24	Center	−774.6
		Lowest Energy	−816.3
13	23	Center	−784.7
		Lowest Energy	−812.1
14	23	Center	−794.1
		Lowest Energy	−823.8
15	22	Center	−769.6
		Lowest Energy	−848.1
16	20	Center	−836.4
		Lowest Energy	−917.3
17	19	Center	−738.3
		Lowest Energy	−827.6
18	18	Center	−863.2
		Lowest Energy	−863.2

Cluster 0, which had the highest number of members (n = 81), showed a comparatively higher weighted score (−848.7 kcal/mol), but it indicates a dense binding mode and is important in terms of statistical reliability. Cluster 16, with a lowest energy of −917.3 kcal/mol, and Cluster 4 (−906.5 kcal/mol) suggest other strong binding orientations, although their center conformations are relatively less stable.

Cluster 18 had evidence of balanced or recurrent interaction, as the values of the center and lowest energy scores were also identical (−863.2 kcal/mol), indicating a stable and reproducibly structured binding interface. The analysis suggests the electrostatic interaction-based docking configuration offers more than one possible binding conformation between HCV Core Protein and IRS-1. The energy scores are marginally greater than the configuration preferred by hydrophobic forces, but numerous clusters, particularly Clusters 5 and 6, show very strong electrostatic anchoring.

## 4. Discussion

This study presents a multi-faceted examination of the association between insulin resistance (IR) and the risk of developing hepatocellular carcinoma (HCC) in subjects with chronic hepatitis C virus (HCV) genotype-3 infection that includes clinical, biochemical, and molecular-level components. It does seem logical that the interaction of HCV core protein with hepatic IRS1 dysregulates insulin signaling. which in turn leads to metabolic dysregulation, fibrosis, and HCC development.

The core protein of the hepatitis C virus (HCV) interacts directly with insulin receptor substrate-1 (IRS-1), a key component of the insulin signaling pathway, to impair its function significantly. The interaction causes the disruption of the insulin signaling pathway, and subsequently, insulin resistance develops. IRS-1 mainly relays messages from the insulin receptor inside the cell. With this message, IRS-1 helps in metabolic regulation [26,27]. The strong downregulation of IRS-1 by infection with hepatitis C virus (HCV) results in hepatic lipid accumulation, oxidative stress, and inflammation. Genetic mutations in the hepatocytes occur due to these modifications that cause the formation of HCC, which is a gradual process. As per studies, different HCV genotypes affect the IRS-1 pathway differently. Genotype 1b causes increased degradation of IRS-1 protein in a direct way [28,29].

The levels of HOMA-IR, fasting insulin, and HbA1c get progressively increased in patients with CHC and HCC, which suggests that glucose homeostasis is dysregulated during the early stages of HCV infection [30,31]. While some of these indicators lie in the statistical reference ranges, their clinical patterns and trajectories suggest a strong causality.

Patients with elevated HOMA-IR (>4) show a strong association with metabolic dysregulation in HCV infection, suggesting that insulin resistance may play an important role in disease progression rather than representing a simple comorbid condition. Previous studies by Bose et al. (2014) and Liu et al. (2024) have also reported that HCV infection is associated with disruption of insulin signaling pathways, particularly involving IRS-1 and AKT signaling [32,33]. which is consistent with the findings of the present study.

However, these studies primarily focused on biochemical and pathway-level associations without directly linking molecular mechanisms to clinical disease progression or incorporating structural interaction analysis. In contrast, the present study integrates clinical outcome-based data with molecular docking evidence, providing additional insight into potential structural interactions between HCV core protein and insulin signaling components. This combined approach offers a more comprehensive perspective on HCV-associated metabolic dysregulation and its possible contribution to disease severity and progression.

Among the various genotypes of HCV, genotype 3 appears to be the most detrimental, as it exerts a direct impact on the insulin signaling pathway [34,35]. Our study found that patients infected with genotype 3 exhibited comparatively higher levels of fasting insulin, HOMA-IR, and HbA1c-all of which were clinically significant.

The results we obtained strongly corroborate those of Hsieh et al. (2012) and Gao et al. (2015), which stated that ‘genotype 3’ inhibits the AKT pathway through the PTEN pathway and also promotes degradation of IRS-1. Consequently, cellular signaling is impaired as insulin receptors can’t transduce the signals anymore [36,37]. In agreement with these studies, our molecular docking analysis demonstrates a strong interaction between the HCV core protein and the PTB domain of IRS-1, indicating a potential structural basis for impairment of IRS-1-mediated signaling. This interaction may contribute to altered insulin signaling cascades involving PI3K/AKT pathways and downstream metabolic regulation.

However, unlike previous studies that primarily focused on biochemical and pathway-level associations, the present study provides additional in silico structural evidence supporting the interaction between HCV genotype 3 core protein and IRS-1, thereby linking molecular interaction patterns with clinically observed insulin resistance and progression toward hepatocellular carcinoma (HCC).

A significant clinical association between insulin resistance and liver fibrosis was observed in this study. Almost all patients with HOMA-IR > 4 presented with stage III–V fibrosis, indicating a close relationship between metabolic dysfunction and fibrotic progression. Insulin resistance may contribute to oxidative stress, chronic inflammation, and hepatocyte injury, which in turn may promote fibrosis progression and increase the risk of hepatic tumor development. Similarly, advanced fibrosis may further impair hepatic insulin signaling, suggesting a potential bidirectional interaction that may contribute to increased disease severity. These findings are consistent with previous studies [30,38–40], which reported that chronic inflammation and insulin resistance jointly contribute to the development of fibrosis and hepatocellular carcinoma (HCC) in HCV infection.

In addition, elevated levels of HOMA-IR, HbA1c, and triglycerides were observed in obese patients. The higher fibrosis stages observed in obese individuals suggest that obesity may exacerbate hepatic insulin resistance and metabolic stress. Although insulin resistance is a well-known consequence of obesity, the presence of non-obese HCC patients suggests that viral and genotype-related factors may also independently impair insulin signaling. Therefore, obesity-related metabolic dysregulation and liver fibrosis may act synergistically to promote disease progression from chronic hepatitis C to hepatocellular carcinoma (HCC) [41–44].

Our computational docking and modeling analyses indicate a potential high-affinity interaction between the HCV core protein and the IRS-1 protein, suggesting a possible influence on the PI3K–AKT signaling pathway. This interaction may reduce AKT activation, which is closely associated with key metabolic processes such as GLUT4 translocation, glycogen synthesis, and glucose uptake. Consequently, this interaction may contribute to the development of hepatic insulin resistance [45–47].

The cluster-based docking energy scores (Cluster 5: −965.8 kcal/mol; Cluster 6: −962.3 kcal/mol) further support the likelihood of a thermodynamically favorable binding interaction between the HCV core protein and IRS-1. Although these findings are based on in silico predictions, they provide a plausible structural basis for IRS-1–mediated disruption of insulin signaling. This potential interaction may contribute to dysregulation of insulin signaling pathways and subsequent metabolic dysfunction [48,49]. However, experimental validation through in vitro and in vivo studies is required to confirm the biological significance of these observations.

This study used a sequential and standard statistical approach to assess the individual effect of several clinical and metabolic indicators. The Mann-Whitney U test and later chi-square test were used to evaluate significant differences among patient groups with carcinoma CHC-HCC and obese patients. In the subsequent phase, univariate logistic regression was used to assess risk factors individually [50–52]. To adjust for confounding variables, we finally applied a multivariate logistic regression model to determine reliable and independent risk factors.

Based on this evidence, covariates such as AST > 40 IU/L and late fibrosis (Stage III-IV) were associated with HCC risk in the current study (statistically significant with a P-value less than 0.05). Every statistical method that was used in the analysis has been chosen based on the research question; as such, the results are therefore credible and scientific [53].

The overall results of the study suggest insulin resistance (IR) is a powerful pathophysiological factor in HCV genotype 3 infection that drives the deterioration of liver function and advanced fibrosis, which ultimately leads to hepatocellular carcinoma (HCC) through HCV core-mediated biochemical impairment of the IRS-1 pathway. Clinical and in silico results show that AST, its HbA1c, AFP, HOMA-IR > 4 and advanced fibrosis stage are independent and strong predictors of HCC development. This study further supports previous studies and established the importance of these markers in genotype 3 infection [30,54,55].

Insulin resistance (IR), even without obesity, could play a salient role in cancer development or carcinogenesis [56]. This finding denotes a direct relationship between the insulin signaling and oncology of virus dynamics. The clinical implication of this study for the future would be the identification of patients at high risk by using markers of insulin resistance and liver fibrosis that would help in the creation of novel therapeutic strategies to target IRS-1. Different approaches could help prevent liver cancer linked to the type 3 virus [26,57].

Despite the strengths of the clinical and in silico findings, this study has several limitations that should be acknowledged when interpreting the results. First, being a single-center and cross-sectional study, the findings may have limited generalizability, and causal relationships between insulin resistance, fibrosis, and HCC progression cannot be established. Therefore, the results should be interpreted as associative rather than causative. Second, metabolic markers such as HOMA-IR and HbA1c were based on clinical measurements, which reflect physiological associations but do not confirm direct mechanistic causation.

In addition, the molecular docking results provide only in silico predictions of protein–protein interactions between the HCV core protein and IRS-1. As these findings have not been validated through in vitro or in vivo experiments, they should be considered hypothesis-generating rather than confirmatory evidence. Future multi-center studies with larger sample sizes and experimental validation are required to strengthen and verify these findings, and to better understand genotype 3-specific viral and host interactions.

## 5. Conclusion

A study identified insulin resistance, advanced fibrosis, and elevated AFP levels as significant risk factors associated with hepatocellular carcinoma (HCC) in patients infected with HCV genotype 3. In addition, in silico docking analysis demonstrated a strong binding interaction between the HCV core protein and IRS-1, suggesting a potential disruption of insulin signaling pathways that may contribute to metabolic dysregulation. These findings indicate that insulin resistance is not only a metabolic disorder but may also act as a key molecular pathway involved in HCV genotype 3-associated HCC through the interaction between HCV core protein and IRS-1. IRS-1 may therefore represent a potential therapeutic target for future research.
