# Supplementary material for: The role of insulin resistance in the development of hepatocellular carcinoma with computational analysis of IRS-1 interaction with HCV genotype 3 core protein
Source: PLoS One. 2026 Sep 25;21(9):e0358858. doi: 10.1371/journal.pone.0358858 (PMC13614604; doi:10.1371/journal.pone.0358858)
Supplement: Supplementary file 1 [file pone.0358858.s002.docx]

**Dear Editor-in-Chief,**

We sincerely thank you for your careful reading of our manuscript *“****The Role of Insulin Resistance in the Development of Hepatocellular Carcinoma with Computational Analysis of IRS-1 Interaction with HCV Genotype 3 Core Protein****”,* EMID:8127de19069f0473, and for your constructive and insightful comments. We appreciate the opportunity to clarify and strengthen the manuscript accordingly. Our point-by-point responses to your comments are provided below.

**Comments 1.**

Please include a complete copy of PLOS’ questionnaire on inclusivity in global research in your revised manuscript. Our policy for research in this area aims to improve transparency in the reporting of research performed outside of researchers’ own country or community. The policy applies to researchers who have travelled to a different country to conduct research, research with Indigenous populations or their lands, and research on cultural artefacts. The questionnaire can also be requested at the journal’s discretion for any other submissions, even if these conditions are not met. Please find more information on the policy and a link to download a blank copy of the questionnaire here: https://journals.plos.org/plosone/s/best-practices-in-research-reporting. Please upload a completed version of your questionnaire as Supporting Information when you resubmit your manuscript.

**Response 1.**

We sincerely thank the editor for this important suggestion. In accordance with PLOS’ policy on inclusivity in global research reporting, we have completed the Inclusivity in Global Research questionnaire and uploaded it as a Supporting Information file during the revised submission. The completed questionnaire provides the requested information regarding the research context, local collaboration, ethical considerations, and stakeholder involvement.

**Comments 2.**

Thank you for updating your data availability statement. You note that your data are available within the Supporting Information files, but no such files have been included with your submission. At this time, we ask that you please upload your minimal data set as a Supporting Information file, or to a public repository such as Figshare or Dryad. Please also ensure that when you upload your file you include separate captions for your supplementary files at the end of your manuscript. As soon as you confirm the location of the data underlying your findings, we will be able to proceed with the review of your submission.

**Response 2.**

We sincerely thank the editor for this important clarification. In the revised manuscript, we have updated the Data Availability Statement to accurately reflect the accessibility of the clinical data. Due to ethical and privacy restrictions, the patient-level clinical data cannot be publicly deposited. However, anonymized data may be made available from the corresponding author upon reasonable request and subject to approval from the relevant institutional ethics committee. **[Line No: 663-666]**

**Comments 3.**

We note that your author list was updated during the revision process. In order to add or remove authors or update the order of the author byline after initial submission, we ask that authors complete an Authorship Change Request form. You may review our full authorship change policy and download the Authorship Change Request form here: <https://journals.plos.org/plosone/s/authorship#loc-authorship-changes>.

**Response 3.**

We sincerely thank the editor for the opportunity to clarify the authorship changes. Following a careful reassessment of each author's contributions according to the PLOS authorship criteria, we determined that Md. Robiul Islam did not meet the required criteria for authorship in the revised manuscript. Therefore, we have removed his name from the author list and updated the Authorship Change Request form accordingly.

**Sincerely,**

**Dr. Mohd Hasan Mujahid**

Post Doctoral Fellow

Department of Polymer & Process Engineering,

Indian Institute of Technology Roorkee, Uttarakhand, INDIA

E-mail: [mhasanmujahid@gmail.com](mailto:mhasanmujahid@gmail.com) ; [mhasan.pd@pe.iitr.ac.in](mailto:mhasan.pd@pe.iitr.ac.in)

Mobile No: +91-9721293563

[Co-corresponding Author]
