# Supplementary material for: The role of insulin resistance in the development of hepatocellular carcinoma with computational analysis of IRS-1 interaction with HCV genotype 3 core protein
Source: PLoS One. 2026 Sep 25;21(9):e0358858. doi: 10.1371/journal.pone.0358858 (PMC13614604; doi:10.1371/journal.pone.0358858)
Supplement: Supplementary file 2 [file pone.0358858.s003.docx]

**Dear Editor-in-Chief,**

We sincerely thank you for your careful reading of our manuscript *“****The Role of Insulin Resistance in the Development of Hepatocellular Carcinoma with Computational Analysis of IRS-1 Interaction with HCV Genotype 3 Core Protein****”,* EMID:8127de19069f0473, and for your constructive and insightful comments. We appreciate the opportunity to clarify and strengthen the manuscript accordingly. Our point-by-point responses to your comments are provided below.

**Abstract**

**Comments 1.**

Only the binding energy value "-1196.0" is provided without specifying the unit. A brief explanation of its biological significance (e.g., "indicating a strong interaction between the two proteins") should be added for clarity.

**Response 1.**

We thank the reviewer for this valuable suggestion. In the revised manuscript, we have specified the unit of the binding energy value as kcal/mol. **[Line No. 35-37]**

**Comments 2.**

The core role of HOMA-IR is not explicitly stated (only numerical values are presented in results). Supplementary description of its association as a key indicator of insulin resistance is needed to align with the listed keywords.

**Response 2.**

In the revised manuscript, we have explicitly clarified the role of HOMA-IR as a key surrogate marker of insulin resistance. It is now described as an important indicator reflecting underlying metabolic dysregulation in HCV-infected patients, thereby improving clarity and ensuring better alignment with the study keywords. **[Line No. 29-31]**

**Introduction**

**Comments 3.**

The statement "most studies have concentrated on genotype 1" lacks elaboration on the core differences between genotype 3 and other genotypes in insulin resistance-related mechanisms (e.g., higher susceptibility to metabolic disorders, faster fibrosis progression). Specific analysis of the research gaps for genotype 3 should be added.

**Response 3.**

The revised manuscript has been updated to clearly describe the differences between HCV genotype 3 and other genotypes, particularly genotype 1, in relation to insulin resistance, hepatic steatosis, metabolic disturbances, and fibrosis progression. Additionally, we have highlighted the limited genotype 3–specific evidence, which has been identified as a key research gap supporting the rationale of this study. **[Line No. 54-60].**

**Comments 4.**

Only two references (1,2) are cited for the claim "HCV genotype 3 is closely associated with hepatic steatosis". Recent (5 years) epidemiological data from South Asian populations or US immigrant groups should be supplemented to strengthen the rationale for this study.

**Response 4.**

We appreciate the reviewer’s valuable suggestion. In the revised manuscript, we have added three additional recent references to strengthen the evidence supporting the association between HCV genotype 3 and hepatic steatosis. These updated citations, including recent epidemiological data from relevant populations, have further reinforced the rationale of the present study. **[Line No. 49]**

**Materials and Methods**

**Comments 5.**

The statistical power calculation process for the 110 samples (20 controls, 45 CHC, 45 HCC) is not described A sample size estimation basis must be supplemented to ensure the scientific rigor of the study design.

**Response 5.**

We thank the reviewer for this valuable comment. In the revised manuscript, we have now provided a clear description of the sample size estimation. The sample size (n=110) was determined based on pilot data and study feasibility, ensuring adequate representation across all clinical groups. Additionally, subgroup analyses were performed (obese vs non-obese), and the sample size was considered to provide approximately 80% statistical power at a significance level of 0.05 to detect clinically meaningful differences in key metabolic and biochemical variables. These details have now been incorporated into the Methods section to improve the transparency and methodological rigor of the study. **[Line No. 196-205]**

**Comments 6.**

The Mann-Whitney U test was used for continuous data, but no verification evidence for non-normal distribution is provided. For multivariate logistic regression analysis, model fit indices are not reported; supplementation is required to evaluate model reliability.

**Response 6.**

We thank the reviewer for this valuable suggestion. In the revised manuscript, the statistical analysis section has been updated to include assessment of normality using the Shapiro–Wilk test. Based on the distribution of the data, appropriate parametric (t-test/ANOVA) and non-parametric (Mann–Whitney U/Kruskal–Wallis) tests were applied, providing clear justification for the use of non-parametric methods (Statistical Analysis section. **[Line No. 207-213]**

Furthermore, model evaluation for multivariate logistic regression has been strengthened by reporting goodness-of-fit measures. Specifically, the Hosmer–Lemeshow test (P > 0.05) and pseudo-R² values have been included to assess model reliability and explanatory power for both advanced fibrosis and hepatocellular carcinoma (HCC). These results are presented in Table 3 **[Line No. 307-309]** and Table 4 **[Line No. 329-331]** of the Results section.

**Comments 7.**

The obese group only includes 36 cases and the non-obese group 54 cases. A statement on whether this sample size meets the statistical power for sub-group analysis should be added.

**Response 7.**

We thank the reviewer for this important suggestion. In the revised manuscript, we have clarified the justification for the subgroup analysis. The comparison between obese (n=36) and non-obese (n=54) participants was based on pilot data and study feasibility. Although these subgroup sizes are relatively modest, they were considered sufficient to detect clinically meaningful differences in key metabolic and biochemical parameters **[Line No. 196-205]**. The statistical power for subgroup analysis was estimated to be approximately 80% at a significance level (α) of 0.05, indicating an acceptable level of power for the analyses performed. These details have now been clearly stated in the Sample Size and Statistical Power section of the manuscript.

**Results**

**Comments 8.**

Incorrect M:F ratio calculations exist in Table 1 (CHC group: 27 males, 18 females, ratio = 1:0.67, originally stated as 01:01.5; HCC group: 33 males, 12 females, ratio = 1:0.36, originally stated as 01:01.8). All erroneous ratio calculations must be corrected.

**Response 8.**

We sincerely thank the reviewer for pointing out this error. We apologize for the mistake in the previously reported M:F ratios in Table 1. The ratios have now been carefully recalculated and corrected in the revised manuscript to ensure accuracy and consistency of the demographic data. **[Line No. 242-245]**

**Comments 9.**

Multiple format errors (e.g., "not significant p=0.17, p=0.64") exist; the unified expression "P=0.17, P=0.64, no statistically significant difference" should be adopted.

**Response 9.**

We sincerely thank the reviewer for this helpful suggestion. In the revised manuscript, all statistical reporting formats have been standardized. The presentation of p-values has been corrected and unified to “P” format.

**Comments 10.**

Figures 1 and 2 are mentioned in the text, but their core trends are not briefly described (e.g., "Figure 1A shows that HOMA-IR increases significantly with the progression of liver fibrosis stage"). Supplementary description linking figures and text is required.

**Response 10.**

We appreciate the reviewer’s valuable suggestion regarding Figure 1A. In response, we have now clearly described the core trend observed in this figure within the Results section. Specifically, we have highlighted the progressive increase in HOMA-IR with advancing liver fibrosis stages, ensuring better clarity and direct linkage between the figure and the text **[Line No. 399-419]**. The interaction between HCV core protein and IRS-1 has now been described in relation to insulin signalling disruption, providing clearer interpretation of the molecular mechanism illustrated in the Figure 2 **[Line No. 422-446]**.

**Discussion**

**Comments 11.**

The core differences between this study and existing research (e.g., Hsieh et al. 2012, Gao et al. 2015) are not clearly distinguished.

**Response 11.**

We appreciate the reviewer’s insightful comment. In the revised manuscript, we have clarified the key differences between the present study and previous literature. While earlier studies (e.g., Hsieh et al., 2012; Gao et al., 2015) mainly focused on biochemical and pathway-level associations of HCV-induced insulin resistance, particularly involving IRS-1 and AKT signalling, they did not directly integrate clinical outcome data with molecular-level structural analysis. In contrast, the present study combines clinical outcome-based findings with molecular docking analysis, thereby providing additional insight into potential structural interactions between HCV core protein and IRS-1 **[Line No. 551-563]**. This integrated approach offers a more comprehensive understanding of HCV genotype 3–associated metabolic dysregulation and its possible contribution to disease progression.

**Comments 12.**

Superficial interpretation of results, for the finding "obese patients had higher IR markers, but CHC-to-HCC progression was more influenced by the combined effects of fibrosis and metabolic dysregulation", the specific biological mechanism of the "synergistic effect" is not explained.

**Response 12.**

We sincerely thank the reviewer for this insightful comment. In the revised manuscript, we have expanded the interpretation of the results to better explain the potential biological basis of the observed synergistic effect. Specifically, we now describe how obesity-associated insulin resistance and liver fibrosis may interact to exacerbate metabolic dysregulation during the progression from chronic hepatitis C (CHC) to hepatocellular carcinoma (HCC) **[Line No. 591-597]**. This is supported by our molecular docking findings, which demonstrate a strong interaction between the HCV core protein and the PTB domain of IRS-1, suggesting a potential structural disruption of IRS-1-mediated insulin signalling **[Line No. 598-610]**.

**Comments 13.**

Only "single-center, cross-sectional study" is mentioned; “Molecular docking results are not validated in vitro”, the impact of this limitation on the conclusions should be stated.

**Response 13.**

We sincerely thank the reviewer for this important suggestion. In the revised manuscript, we have clarified the impact of these limitations on the interpretation of our findings. Specifically, we acknowledge that being a single-center, cross-sectional study, the results should be interpreted as associative rather than causal, and the generalizability of the findings may be limited **[Line No. 634-646]**. Furthermore, we emphasize that the molecular docking results are in silico predictions and have not been validated experimentally; therefore, they should be considered as hypothesis-generating evidence rather than definitive confirmation of biological mechanisms.

**Comments 14.**

Studies such as Bose et al. 2014 and Liu et al. 2024 are cited, but their consistency/inconsistency with the findings of this study is not explicitly stated. Comparative analysis should be added.

**Response 14.**

We thank the reviewer for this valuable suggestion. In the revised manuscript, we have clarified the comparative interpretation of previous studies with the present findings. Specifically, studies by Bose et al. (2014) and Liu et al. (2024) reported that HCV infection is associated with disruption of insulin signalling pathways, particularly involving IRS-1 and AKT signalling, which is consistent with the findings of the present study **[Line No. 651-556]**. However, while these studies primarily focused on biochemical and pathway-level associations, our study further integrates clinical outcome-based analysis with molecular docking evidence, providing additional structural insight into HCV-related insulin resistance.

**Conclusion**

**Comments 15.**

The existing conclusion only summarizes risk factors and molecular mechanisms, without highlighting the core innovations of the study.

**Response 15.**

We thank the reviewer for this valuable suggestion. In the revised manuscript, we have revised the conclusion to better emphasize the core innovation of the study. Specifically, we highlight that, in addition to identifying insulin resistance, advanced fibrosis, and elevated AFP as key risk factors for HCV genotype 3-associated hepatocellular carcinoma (HCC), the study uniquely integrates clinical outcome data with in silico molecular docking analysis. This combined approach provides additional structural insight into the interaction between HCV core protein and IRS-1, thereby offering a more comprehensive understanding of genotype 3-associated metabolic dysregulation and disease progression **[Line No. 648-655]**.

**Comments 16.**

The statement "IR is not simply a metabolic disorder and may also be a molecular pathway" is overly absolute and should be revised to: "IR is not only a metabolic disorder but is more likely to act as a key molecular pathway for HCV genotype 3-associated HCC through the interaction between HCV core protein and IRS-1" to maintain rigor based on existing results.

**Response 16.**

We sincerely thank the reviewer for this important suggestion. The statement has been revised in the manuscript to ensure scientific rigor and appropriate caution. We now clarify that insulin resistance is not only a metabolic disorder but may more likely act as a key molecular pathway in HCV genotype 3-associated hepatocellular carcinoma through the interaction between HCV core protein and IRS-1 **[Line No. 648-655]**.

**Sincerely,**

**Dr. Mohd Hasan Mujahid**

Post Doctoral Fellow

Department of Polymer & Process Engineering,

Indian Institute of Technology Roorkee, Uttarakhand, INDIA

E-mail: [mhasanmujahid@gmail.com](mailto:mhasanmujahid@gmail.com) ; [mhasan.pd@pe.iitr.ac.in](mailto:mhasan.pd@pe.iitr.ac.in)

Mobile No: +91-9721293563

[Co-corresponding Author]
