# Supplementary material for: The role of insulin resistance in the development of hepatocellular carcinoma with computational analysis of IRS-1 interaction with HCV genotype 3 core protein
Source: PLoS One. 2026 Sep 25;21(9):e0358858. doi: 10.1371/journal.pone.0358858 (PMC13614604; doi:10.1371/journal.pone.0358858)
Supplement: Peer review history

# Peer review: Decision Letter 0

**DOI:** 10.1371/journal.pone.0358858.r001

**Authors:** Yury E Khudyakov

4 Jun 2026

Dear Dr. Mujahid,

Thank you for submitting your manuscript to PLOS ONE. After careful consideration, we feel that it has merit but does not fully meet PLOS ONE’s publication criteria as it currently stands. Therefore, we invite you to submit a revised version of the manuscript that addresses the points raised during the review process.

==============================

Your manuscript was reviewed by one expert in the field who identified several important problems in your submission.  Please review the attached comments and provide point-by-point responses.

==============================

Please submit your revised manuscript by Jul 19 2026 11:59PM. If you will need more time than this to complete your revisions, please reply to this message or contact the journal office at plosone@plos.org. When you're ready to submit your revision, log on to https://www.editorialmanager.com/pone/ and select the 'Submissions Needing Revision' folder to locate your manuscript file.

- A letter that responds to each point raised by the academic editor and reviewer(s). You should upload this letter as a separate file labeled 'Response to Reviewers'.
- A marked-up copy of your manuscript that highlights changes made to the original version. You should upload this as a separate file labeled 'Revised Manuscript with Track Changes'.
- An unmarked version of your revised paper without tracked changes. You should upload this as a separate file labeled 'Manuscript'.

If you would like to make changes to your financial disclosure, please include your updated statement in your cover letter. Guidelines for resubmitting your figure files are available below the reviewer comments at the end of this letter.

If applicable, we recommend that you deposit your laboratory protocols in protocols.io to enhance the reproducibility of your results. Protocols.io assigns your protocol its own identifier (DOI) so that it can be cited independently in the future. For instructions see: https://journals.plos.org/plosone/s/submission-guidelines#loc-laboratory-protocols. Additionally, PLOS ONE offers an option for publishing peer-reviewed Lab Protocol articles, which describe protocols hosted on protocols.io. Read more information on sharing protocols at https://plos.org/protocols?utm_medium=editorial-email&utm_source=authorletters&utm_campaign=protocols.

As the corresponding author, your ORCID iD is verified in the submission system and will appear in the published article. PLOS supports the use of ORCID, and we encourage all coauthors to register for an ORCID iD and use it as well. Please encourage your coauthors to verify their ORCID iD within the submission system before final acceptance, as unverified ORCID iDs will not appear in the published article. *Only*  the individual author can complete the verification step; PLOS staff *cannot*  verify ORCID iDs on behalf of authors.

We look forward to receiving your revised manuscript.

Kind regards,

Yury E Khudyakov, PhD

Academic Editor

PLOS One

Journal Requirements:

When submitting your revision, we need you to address these additional requirements.

1. Please ensure that your manuscript meets PLOS ONE's style requirements, including those for file naming. The PLOS ONE style templates can be found at

https://journals.plos.org/plosone/s/file?id=wjVg/PLOSOne_formatting_sample_main_body.pdf and

https://journals.plos.org/plosone/s/file?id=ba62/PLOSOne_formatting_sample_title_authors_affiliations.pdf

2. Please include a complete copy of PLOS’ questionnaire on inclusivity in global research in your revised manuscript. Our policy for research in this area aims to improve transparency in the reporting of research performed outside of researchers’ own country or community. The policy applies to researchers who have travelled to a different country to conduct research, research with Indigenous populations or their lands, and research on cultural artefacts. The questionnaire can also be requested at the journal’s discretion for any other submissions, even if these conditions are not met.  Please find more information on the policy and a link to download a blank copy of the questionnaire here: https://journals.plos.org/plosone/s/best-practices-in-research-reporting. Please upload a completed version of your questionnaire as Supporting Information when you resubmit your manuscript.

3. Please note that PLOS One has specific guidelines on code sharing for submissions in which author-generated code underpins the findings in the manuscript. In these cases, we expect all author-generated code to be made available without restrictions upon publication of the work. Please review our guidelines at https://journals.plos.org/plosone/s/materials-and-software-sharing#loc-sharing-code and ensure that your code is shared in a way that follows best practice and facilitates reproducibility and reuse.

4. We note that your Data Availability Statement is currently as follows: “All relevant data are within the manuscript and its Supporting Information files.”

Please confirm at this time whether or not your submission contains all raw data required to replicate the results of your study. Authors must share the “minimal data set” for their submission. PLOS defines the minimal data set to consist of the data required to replicate all study findings reported in the article, as well as related metadata and methods (https://journals.plos.org/plosone/s/data-availability#loc-minimal-data-set-definition).

For example, authors should submit the following data:

- The values behind the means, standard deviations and other measures reported;

- The values used to build graphs;

- The points extracted from images for analysis.

Authors do not need to submit their entire data set if only a portion of the data was used in the reported study.

If your submission does not contain these data, please either upload them as Supporting Information files or deposit them to a stable, public repository and provide us with the relevant URLs, DOIs, or accession numbers. For a list of recommended repositories, please see https://journals.plos.org/plosone/s/recommended-repositories.

If there are ethical or legal restrictions on sharing a de-identified data set, please explain them in detail (e.g., data contain potentially sensitive information, data are owned by a third-party organization, etc.) and who has imposed them (e.g., an ethics committee). Please also provide contact information for a data access committee, ethics committee, or other institutional body to which data requests may be sent. If data are owned by a third party, please indicate how others may request data access.

5. Please upload a new copy of Figures 1 and 2 as the detail is not clear. Please follow the link for more information:  https://journals.plos.org/plosone/s/figures

6. If the reviewer comments include a recommendation to cite specific previously published works, please review and evaluate these publications to determine whether they are relevant and should be cited. There is no requirement to cite these works unless the editor has indicated otherwise.

7. Please review your reference list to ensure that it is complete and correct. If you have cited papers that have been retracted, please include the rationale for doing so in the manuscript text, or remove these references and replace them with relevant current references. Any changes to the reference list should be mentioned in the rebuttal letter that accompanies your revised manuscript. If you need to cite a retracted article, indicate the article’s retracted status in the References list and also include a citation and full reference for the retraction notice.

[Note: HTML markup is below. Please do not edit.]

Reviewers' comments:

Reviewer's Responses to Questions

**Comments to the Author**

1. Is the manuscript technically sound, and do the data support the conclusions?

Reviewer #1: Yes

**********

2. Has the statistical analysis been performed appropriately and rigorously?

Reviewer #1: Yes

**********

3. Have the authors made all data underlying the findings in their manuscript fully available?

The PLOS Data policy

Reviewer #1: Yes

**********

4. Is the manuscript presented in an intelligible fashion and written in standard English?

Reviewer #1: Yes

**********

Reviewer #1: Reviewer Recommendation and Comments for Manuscript Number PONE-D-26-01567

Abstracts

1.Only the binding energy value "-1196.0" is provided without specifying the unit. A brief explanation of its biological significance (e.g., "indicating a strong interaction between the two proteins") should be added for clarity.

2.The core role of HOMA-IR is not explicitly stated (only numerical values are presented in results). Supplementary description of its association as a key indicator of insulin resistance is needed to align with the listed keywords.

Introduction

3.The statement "most studies have concentrated on genotype 1" lacks elaboration on the core differences between genotype 3 and other genotypes in insulin resistance-related mechanisms (e.g., higher susceptibility to metabolic disorders, faster fibrosis progression). Specific analysis of the research gaps for genotype 3 should be added.

4.Only two references (1,2) are cited for the claim "HCV genotype 3 is closely associated with hepatic steatosis". Recent (5 years) epidemiological data from South Asian populations or US immigrant groups should be supplemented to strengthen the rationale for this study.

Materials and Methods

5.The statistical power calculation process for the 110 samples (20 controls, 45 CHC, 45 HCC) is not described A sample size estimation basis must be supplemented to ensure the scientific rigor of the study design.

6.The Mann-Whitney U test was used for continuous data, but no verification evidence for non-normal distribution is provided. For multivariate logistic regression analysis, model fit indices are not reported; supplementation is required to evaluate model reliability.

7.The obese group only includes 36 cases and the non-obese group 54 cases. A statement on whether this sample size meets the statistical power for sub-group analysis should be added.

Results

8.Incorrect M:F ratio calculations exist in Table 1 (CHC group: 27 males, 18 females, ratio = 1:0.67, originally stated as 01:01.5; HCC group: 33 males, 12 females, ratio = 1:0.36, originally stated as 01:01.8). All erroneous ratio calculations must be corrected.

9.Multiple format errors (e.g., "not significant p=0.17, p=0.64") exist; the unified expression "P=0.17, P=0.64, no statistically significant difference" should be adopted.

10.Figures 1 and 2 are mentioned in the text, but their core trends are not briefly described (e.g., "Figure 1A shows that HOMA-IR increases significantly with the progression of liver fibrosis stage"). Supplementary description linking figures and text is required.

Discussion

11.The core differences between this study and existing research (e.g., Hsieh et al. 2012, Gao et al. 2015) are not clearly distinguished.

12.Superficial interpretation of results, for the finding "obese patients had higher IR markers, but CHC-to-HCC progression was more influenced by the combined effects of fibrosis and metabolic dysregulation", the specific biological mechanism of the "synergistic effect" is not explained.

13.Only "single-center, cross-sectional study" is mentioned; “Molecular docking results are not validated in vitro” , the impact of this limitation on the conclusions should be stated.

14.Studies such as Bose et al. 2014 and Liu et al. 2024 are cited, but their consistency/inconsistency with the findings of this study is not explicitly stated. Comparative analysis should be added.

Conclusion

15. The existing conclusion only summarizes risk factors and molecular mechanisms, without highlighting the core innovations of the study.

16.The statement "IR is not simply a metabolic disorder and may also be a molecular pathway" is overly absolute and should be revised to: "IR is not only a metabolic disorder but is more likely to act as a key molecular pathway for HCV genotype 3-associated HCC through the interaction between HCV core protein and IRS-1" to maintain rigor based on existing results.

**********

what does this mean?). If published, this will include your full peer review and any attached files.

If you choose “no”, your identity will remain anonymous but your review may still be made public.

**Do you want your identity to be public for this peer review?**  For information about this choice, including consent withdrawal, please see our Privacy Policy

Reviewer #1: No

**********

[NOTE: If reviewer comments were submitted as an attachment file, they will be attached to this email and accessible via the submission site. Please log into your account, locate the manuscript record, and check for the action link "View Attachments". If this link does not appear, there are no attachment files.]

To ensure your figures meet our technical requirements, please review our figure guidelines: https://journals.plos.org/plosone/s/figures

You may also use PLOS’s free figure tool, NAAS, to help you prepare publication quality figures: https://journals.plos.org/plosone/s/figures#loc-tools-for-figure-preparation.

NAAS will assess whether your figures meet our technical requirements by comparing each figure against our figure specifications.

# Author comment: Author response to Decision Letter 1

**DOI:** 10.1371/journal.pone.0358858.r002

9 Aug 2026

Dear Editor-in-Chief,

We sincerely thank you for your careful reading of our manuscript “The Role of Insulin Resistance in the Development of Hepatocellular Carcinoma with Computational Analysis of IRS-1 Interaction with HCV Genotype 3 Core Protein”, EMID:8127de19069f0473, and for your constructive and insightful comments. We appreciate the opportunity to clarify and strengthen the manuscript accordingly. Our point-by-point responses to your comments are provided below.

Comments 1.

Please include a complete copy of PLOS’ questionnaire on inclusivity in global research in your revised manuscript. Our policy for research in this area aims to improve transparency in the reporting of research performed outside of researchers’ own country or community. The policy applies to researchers who have travelled to a different country to conduct research, research with Indigenous populations or their lands, and research on cultural artefacts. The questionnaire can also be requested at the journal’s discretion for any other submissions, even if these conditions are not met. Please find more information on the policy and a link to download a blank copy of the questionnaire here: https://journals.plos.org/plosone/s/best-practices-in-research-reporting. Please upload a completed version of your questionnaire as Supporting Information when you resubmit your manuscript.

Response 1.

We sincerely thank the editor for this important suggestion. In accordance with PLOS’ policy on inclusivity in global research reporting, we have completed the Inclusivity in Global Research questionnaire and uploaded it as a Supporting Information file during the revised submission. The completed questionnaire provides the requested information regarding the research context, local collaboration, ethical considerations, and stakeholder involvement.

Comments 2.

Thank you for updating your data availability statement. You note that your data are available within the Supporting Information files, but no such files have been included with your submission. At this time, we ask that you please upload your minimal data set as a Supporting Information file, or to a public repository such as Figshare or Dryad. Please also ensure that when you upload your file you include separate captions for your supplementary files at the end of your manuscript. As soon as you confirm the location of the data underlying your findings, we will be able to proceed with the review of your submission.

Response 2.

We sincerely thank the editor for this important clarification. In the revised manuscript, we have updated the Data Availability Statement to accurately reflect the accessibility of the clinical data. Due to ethical and privacy restrictions, the patient-level clinical data cannot be publicly deposited. However, anonymized data may be made available from the corresponding author upon reasonable request and subject to approval from the relevant institutional ethics committee. [Line No: 663-666]

Comments 3.

We note that your author list was updated during the revision process. In order to add or remove authors or update the order of the author byline after initial submission, we ask that authors complete an Authorship Change Request form. You may review our full authorship change policy and download the Authorship Change Request form here: https://journals.plos.org/plosone/s/authorship#loc-authorship-changes.

Response 3.

We sincerely thank the editor for the opportunity to clarify the authorship changes. Following a careful reassessment of each author's contributions according to the PLOS authorship criteria, we determined that Md. Robiul Islam did not meet the required criteria for authorship in the revised manuscript. Therefore, we have removed his name from the author list and updated the Authorship Change Request form accordingly.

Sincerely,

Dr. Mohd Hasan Mujahid

Post Doctoral Fellow

Department of Polymer & Process Engineering,

Indian Institute of Technology Roorkee, Uttarakhand, INDIA

E-mail: mhasanmujahid@gmail.com ; mhasan.pd@pe.iitr.ac.in

Mobile No: +91-9721293563

[Corresponding Author]

Attachment Submitted filename: Rebuttal letter.docx

# Editor report: Decision Letter 1

**DOI:** 10.1371/journal.pone.0358858.r003

**Authors:** Yury E Khudyakov

16 Aug 2026

Dear Dr. Mujahid,

Thank you for submitting your manuscript to PLOS ONE. After careful consideration, we feel that it has merit but does not fully meet PLOS ONE’s publication criteria as it currently stands. Therefore, we invite you to submit a revised version of the manuscript that addresses the points raised during the review process.

==============================

Abstracts

1.Only the binding energy value "-1196.0" is provided without specifying the unit. A brief explanation of its biological significance (e.g., "indicating a strong interaction between the two proteins") should be added for clarity.

2.The core role of HOMA-IR is not explicitly stated (only numerical values are presented in results). Supplementary description of its association as a key indicator of insulin resistance is needed to align with the listed keywords.

Introduction

3.The statement "most studies have concentrated on genotype 1" lacks elaboration on the core differences between genotype 3 and other genotypes in insulin resistance-related mechanisms (e.g., higher susceptibility to metabolic disorders, faster fibrosis progression). Specific analysis of the research gaps for genotype 3 should be added.

4.Only two references (1,2) are cited for the claim "HCV genotype 3 is closely associated with hepatic steatosis". Recent (5 years) epidemiological data from South Asian populations or US immigrant groups should be supplemented to strengthen the rationale for this study.

Materials and Methods

5.The statistical power calculation process for the 110 samples (20 controls, 45 CHC, 45 HCC) is not described A sample size estimation basis must be supplemented to ensure the scientific rigor of the study design.

6.The Mann-Whitney U test was used for continuous data, but no verification evidence for non-normal distribution is provided. For multivariate logistic regression analysis, model fit indices are not reported; supplementation is required to evaluate model reliability.

7.The obese group only includes 36 cases and the non-obese group 54 cases. A statement on whether this sample size meets the statistical power for sub-group analysis should be added.

Results

8.Incorrect M:F ratio calculations exist in Table 1 (CHC group: 27 males, 18 females, ratio = 1:0.67, originally stated as 01:01.5; HCC group: 33 males, 12 females, ratio = 1:0.36, originally stated as 01:01.8). All erroneous ratio calculations must be corrected.

9.Multiple format errors (e.g., "not significant p=0.17, p=0.64") exist; the unified expression "P=0.17, P=0.64, no statistically significant difference" should be adopted.

10.Figures 1 and 2 are mentioned in the text, but their core trends are not briefly described (e.g., "Figure 1A shows that HOMA-IR increases significantly with the progression of liver fibrosis stage"). Supplementary description linking figures and text is required.

Discussion

11.The core differences between this study and existing research (e.g., Hsieh et al. 2012, Gao et al. 2015) are not clearly distinguished.

12.Superficial interpretation of results, for the finding "obese patients had higher IR markers, but CHC-to-HCC progression was more influenced by the combined effects of fibrosis and metabolic dysregulation", the specific biological mechanism of the "synergistic effect" is not explained.

13.Only "single-center, cross-sectional study" is mentioned; “Molecular docking results are not validated in vitro” , the impact of this limitation on the conclusions should be stated.

14.Studies such as Bose et al. 2014 and Liu et al. 2024 are cited, but their consistency/inconsistency with the findings of this study is not explicitly stated. Comparative analysis should be added.

Conclusion

15. The existing conclusion only summarizes risk factors and molecular mechanisms, without highlighting the core innovations of the study.

16.The statement "IR is not simply a metabolic disorder and may also be a molecular pathway" is overly absolute and should be revised to: "IR is not only a metabolic disorder but is more likely to act as a key molecular pathway for HCV genotype 3-associated HCC through the interaction between HCV core protein and IRS-1" to maintain rigor based on existing results.

==============================

Please submit your revised manuscript by Oct 14 2026 11:59PM. If you will need more time than this to complete your revisions, please reply to this message or contact the journal office at plosone@plos.org. When you're ready to submit your revision, log on to https://www.editorialmanager.com/pone/ and select the 'Submissions Needing Revision' folder to locate your manuscript file.

- A letter that responds to each point raised by the academic editor and reviewer(s). You should upload this letter as a separate file labeled 'Response to Reviewers'.
- A marked-up copy of your manuscript that highlights changes made to the original version. You should upload this as a separate file labeled 'Revised Manuscript with Track Changes'.
- An unmarked version of your revised paper without tracked changes. You should upload this as a separate file labeled 'Manuscript'.

If you would like to make changes to your financial disclosure, please include your updated statement in your cover letter. Guidelines for resubmitting your figure files are available below the reviewer comments at the end of this letter.

If applicable, we recommend that you deposit your laboratory protocols in protocols.io to enhance the reproducibility of your results. Protocols.io assigns your protocol its own identifier (DOI) so that it can be cited independently in the future. For instructions see: https://journals.plos.org/plosone/s/submission-guidelines#loc-laboratory-protocols. Additionally, PLOS ONE offers an option for publishing peer-reviewed Lab Protocol articles, which describe protocols hosted on protocols.io. Read more information on sharing protocols at https://plos.org/protocols?utm_medium=editorial-email&utm_source=authorletters&utm_campaign=protocols.

As the corresponding author, your ORCID iD is verified in the submission system and will appear in the published article. PLOS supports the use of ORCID, and we encourage all coauthors to register for an ORCID iD and use it as well. Please encourage your coauthors to verify their ORCID iD within the submission system before final acceptance, as unverified ORCID iDs will not appear in the published article. *Only*  the individual author can complete the verification step; PLOS staff *cannot*  verify ORCID iDs on behalf of authors.

We look forward to receiving your revised manuscript.

Kind regards,

Yury E Khudyakov, PhD

Academic Editor

PLOS One

Journal Requirements:

If the reviewer comments include a recommendation to cite specific previously published works, please review and evaluate these publications to determine whether they are relevant and should be cited. There is no requirement to cite these works unless the editor has indicated otherwise.

Please review your reference list to ensure that it is complete and correct. If you have cited papers that have been retracted, please include the rationale for doing so in the manuscript text, or remove these references and replace them with relevant current references. Any changes to the reference list should be mentioned in the rebuttal letter that accompanies your revised manuscript. If you need to cite a retracted article, indicate the article’s retracted status in the References list and also include a citation and full reference for the retraction notice.

[Note: HTML markup is below. Please do not edit.]

[NOTE: If reviewer comments were submitted as an attachment file, they will be attached to this email and accessible via the submission site. Please log into your account, locate the manuscript record, and check for the action link "View Attachments". If this link does not appear, there are no attachment files.]

To ensure your figures meet our technical requirements, please review our figure guidelines: https://journals.plos.org/plosone/s/figures

You may also use PLOS’s free figure tool, NAAS, to help you prepare publication quality figures: https://journals.plos.org/plosone/s/figures#loc-tools-for-figure-preparation.

NAAS will assess whether your figures meet our technical requirements by comparing each figure against our figure specifications.

# Author comment: Author response to Decision Letter 2

**DOI:** 10.1371/journal.pone.0358858.r004

4 Sep 2026

Dear Editor-in-Chief,

We sincerely thank you for your careful reading of our manuscript “The Role of Insulin Resistance in the Development of Hepatocellular Carcinoma with Computational Analysis of IRS-1 Interaction with HCV Genotype 3 Core Protein”, EMID:8127de19069f0473, and for your constructive and insightful comments. We appreciate the opportunity to clarify and strengthen the manuscript accordingly. Our point-by-point responses to your comments are provided below.

Abstract

Comments 1.

Only the binding energy value "-1196.0" is provided without specifying the unit. A brief explanation of its biological significance (e.g., "indicating a strong interaction between the two proteins") should be added for clarity.

Response 1.

We thank the reviewer for this valuable suggestion. In the revised manuscript, we have specified the unit of the binding energy value as kcal/mol. [Line No. 35-37]

Comments 2.

The core role of HOMA-IR is not explicitly stated (only numerical values are presented in results). Supplementary description of its association as a key indicator of insulin resistance is needed to align with the listed keywords.

Response 2.

In the revised manuscript, we have explicitly clarified the role of HOMA-IR as a key surrogate marker of insulin resistance. It is now described as an important indicator reflecting underlying metabolic dysregulation in HCV-infected patients, thereby improving clarity and ensuring better alignment with the study keywords. [Line No. 29-31]

Introduction

Comments 3.

The statement "most studies have concentrated on genotype 1" lacks elaboration on the core differences between genotype 3 and other genotypes in insulin resistance-related mechanisms (e.g., higher susceptibility to metabolic disorders, faster fibrosis progression). Specific analysis of the research gaps for genotype 3 should be added.

Response 3.

The revised manuscript has been updated to clearly describe the differences between HCV genotype 3 and other genotypes, particularly genotype 1, in relation to insulin resistance, hepatic steatosis, metabolic disturbances, and fibrosis progression. Additionally, we have highlighted the limited genotype 3–specific evidence, which has been identified as a key research gap supporting the rationale of this study. [Line No. 54-60].

Comments 4.

Only two references (1,2) are cited for the claim "HCV genotype 3 is closely associated with hepatic steatosis". Recent (5 years) epidemiological data from South Asian populations or US immigrant groups should be supplemented to strengthen the rationale for this study.

Response 4.

We appreciate the reviewer’s valuable suggestion. In the revised manuscript, we have added three additional recent references to strengthen the evidence supporting the association between HCV genotype 3 and hepatic steatosis. These updated citations, including recent epidemiological data from relevant populations, have further reinforced the rationale of the present study. [Line No. 49]

Materials and Methods

Comments 5.

The statistical power calculation process for the 110 samples (20 controls, 45 CHC, 45 HCC) is not described A sample size estimation basis must be supplemented to ensure the scientific rigor of the study design.

Response 5.

We thank the reviewer for this valuable comment. In the revised manuscript, we have now provided a clear description of the sample size estimation. The sample size (n=110) was determined based on pilot data and study feasibility, ensuring adequate representation across all clinical groups. Additionally, subgroup analyses were performed (obese vs non-obese), and the sample size was considered to provide approximately 80% statistical power at a significance level of 0.05 to detect clinically meaningful differences in key metabolic and biochemical variables. These details have now been incorporated into the Methods section to improve the transparency and methodological rigor of the study. [Line No. 196-205]

Comments 6.

The Mann-Whitney U test was used for continuous data, but no verification evidence for non-normal distribution is provided. For multivariate logistic regression analysis, model fit indices are not reported; supplementation is required to evaluate model reliability.

Response 6.

We thank the reviewer for this valuable suggestion. In the revised manuscript, the statistical analysis section has been updated to include assessment of normality using the Shapiro–Wilk test. Based on the distribution of the data, appropriate parametric (t-test/ANOVA) and non-parametric (Mann–Whitney U/Kruskal–Wallis) tests were applied, providing clear justification for the use of non-parametric methods (Statistical Analysis section. [Line No. 207-213]

Furthermore, model evaluation for multivariate logistic regression has been strengthened by reporting goodness-of-fit measures. Specifically, the Hosmer–Lemeshow test (P > 0.05) and pseudo-R² values have been included to assess model reliability and explanatory power for both advanced fibrosis and hepatocellular carcinoma (HCC). These results are presented in Table 3 [Line No. 307-309] and Table 4 [Line No. 329-331] of the Results section.

Comments 7.

The obese group only includes 36 cases and the non-obese group 54 cases. A statement on whether this sample size meets the statistical power for sub-group analysis should be added.

Response 7.

We thank the reviewer for this important suggestion. In the revised manuscript, we have clarified the justification for the subgroup analysis. The comparison between obese (n=36) and non-obese (n=54) participants was based on pilot data and study feasibility. Although these subgroup sizes are relatively modest, they were considered sufficient to detect clinically meaningful differences in key metabolic and biochemical parameters [Line No. 196-205]. The statistical power for subgroup analysis was estimated to be approximately 80% at a significance level (α) of 0.05, indicating an acceptable level of power for the analyses performed. These details have now been clearly stated in the Sample Size and Statistical Power section of the manuscript.

Results

Comments 8.

Incorrect M:F ratio calculations exist in Table 1 (CHC group: 27 males, 18 females, ratio = 1:0.67, originally stated as 01:01.5; HCC group: 33 males, 12 females, ratio = 1:0.36, originally stated as 01:01.8). All erroneous ratio calculations must be corrected.

Response 8.

We sincerely thank the reviewer for pointing out this error. We apologize for the mistake in the previously reported M:F ratios in Table 1. The ratios have now been carefully recalculated and corrected in the revised manuscript to ensure accuracy and consistency of the demographic data. [Line No. 242-245]

Comments 9.

Multiple format errors (e.g., "not significant p=0.17, p=0.64") exist; the unified expression "P=0.17, P=0.64, no statistically significant difference" should be adopted.

Response 9.

We sincerely thank the reviewer for this helpful suggestion. In the revised manuscript, all statistical reporting formats have been standardized. The presentation of p-values has been corrected and unified to “P” format.

Comments 10.

Figures 1 and 2 are mentioned in the text, but their core trends are not briefly described (e.g., "Figure 1A shows that HOMA-IR increases significantly with the progression of liver fibrosis stage"). Supplementary description linking figures and text is required.

Response 10.

We appreciate the reviewer’s valuable suggestion regarding Figure 1A. In response, we have now clearly described the core trend observed in this figure within the Results section. Specifically, we have highlighted the progressive increase in HOMA-IR with advancing liver fibrosis stages, ensuring better clarity and direct linkage between the figure and the text [Line No. 399-419]. The interaction between HCV core protein and IRS-1 has now been described in relation to insulin signalling disruption, providing clearer interpretation of the molecular mechanism illustrated in the Figure 2 [Line No. 422-446].

Discussion

Comments 11.

The core differences between this study and existing research (e.g., Hsieh et al. 2012, Gao et al. 2015) are not clearly distinguished.

Response 11.

We appreciate the reviewer’s insightful comment. In the revised manuscript, we have clarified the key differences between the present study and previous literature. While earlier studies (e.g., Hsieh et al., 2012; Gao et al., 2015) mainly focused on biochemical and pathway-level associations of HCV-induced insulin resistance, particularly involving IRS-1 and AKT signalling, they did not directly integrate clinical outcome data with molecular-level structural analysis. In contrast, the present study combines clinical outcome-based findings with molecular docking analysis, thereby providing additional insight into potential structural interactions between HCV core protein and IRS-1 [Line No. 551-563]. This integrated approach offers a more comprehensive understanding of HCV genotype 3–associated metabolic dysregulation and its possible contribution to disease progression.

Comments 12.

Superficial interpretation of results, for the finding "obese patients had higher IR markers, but CHC-to-HCC progression was more influenced by the combined effects of fibrosis and metabolic dysregulation", the specific biological mechanism of the "synergistic effect" is not explained.

Response 12.

We sincerely thank the reviewer for this insightful comment. In the revised manuscript, we have expanded the interpretation of the results to better explain the potential biological basis of the observed synergistic effect. Specifically, we now describe how obesity-associated insulin resistance and liver fibrosis may interact to exacerbate metabolic dysregulation during the progression from chronic hepatitis C (CHC) to hepatocellular carcinoma (HCC) [Line No. 591-597]. This is supported by our molecular docking findings, which demonstrate a strong interaction between the HCV core protein and the PTB domain of IRS-1, suggesting a potential structural disruption of IRS-1-mediated insulin signalling [Line No. 598-610].

Comments 13.

Only "single-center, cross-sectional study" is mentioned; “Molecular docking results are not validated in vitro”, the impact of this limitation on the conclusions should be stated.

Response 13.

We sincerely thank the reviewer for this important suggestion. In the revised manuscript, we have clarified the impact of these limitations on the interpretation of our findings. Specifically, we acknowledge that being a single-center, cross-sectional study, the results should be interpreted as associative rather than causal, and the generalizability of the findings may be limited [Line No. 634-646]. Furthermore, we emphasize that the molecular docking results are in silico predictions and have not been validated experimentally; therefore, they should be considered as hypothesis-generating evidence rather than definitive confirmation of biological mechanisms.

Comments 14.

Studies such as Bose et al. 2014 and Liu et al. 2024 are cited, but their consistency/inconsistency with the findings of this study is not explicitly stated. Comparative analysis should be added.

Response 14.

We thank the reviewer for this valuable suggestion. In the revised manuscript, we have clarified the comparative interpretation of previous studies with the present findings. Specifically, studies by Bose et al. (2014) and Liu et al. (2024) reported that HCV infection is associated with disruption of insulin signalling pathways, particularly involving IRS-1 and AKT signalling, which is consistent with the findings of the present study [Line No. 651-556]. However, while these studies primarily focused on biochemical and pathway-level associations, our study further integrates clinical outcome-based analysis with molecular docking evidence, providing additional structural insight into HCV-related insulin resistance.

Conclusion

Comments 15.

The existing conclusion only summarizes risk factors and molecular mechanisms, without highlighting the core innovations of the study.

Response 15.

We thank the reviewer for this valuable suggestion. In the revised manuscript, we have revised the conclusion to better emphasize the core innovation of the study. Specifically, we highlight that, in addition to identifying insulin resistance, advanced fibrosis, and elevated AFP as key risk factors for HCV genotype 3-associated hepatocellular carcinoma (HCC), the study uniquely integrates clinical outcome data with in silico molecular docking analysis. This combined approach provides additional structural insight into the interaction between HCV core protein and IRS-1, thereby offering a more comprehensive understanding of genotype 3-associated metabolic dysregulation and disease progression [Line No. 648-655].

Comments 16.

The statement "IR is not simply a metabolic disorder and may also be a molecular pathway" is overly absolute and should be revised to: "IR is not only a metabolic disorder but is more likely to act as a key molecular pathway for HCV genotype 3-associated HCC through the interaction between HCV core protein and IRS-1" to maintain rigor based on existing results.

Response 16.

We sincerely thank the reviewer for this important suggestion. The statement has been revised in the manuscript to ensure scientific rigor and appropriate caution. We now clarify that insulin resistance is not only a metabolic disorder but may more likely act as a key molecular pathway in HCV genotype 3-associated hepatocellular carcinoma through the interaction between HCV core protein and IRS-1 [Line No. 648-655].

Sincerely,

Dr. Mohd Hasan Mujahid

Post Doctoral Fellow

Department of Polymer & Process Engineering,

Indian Institute of Technology Roorkee, Uttarakhand, INDIA

E-mail: mhasanmujahid@gmail.com ; mhasan.pd@pe.iitr.ac.in

Mobile No: +91-9721293563

[Co-corresponding Author]

Attachment Submitted filename: Rebuttal_letter_auresp_2.docx

# Editor report: Decision Letter 2

**DOI:** 10.1371/journal.pone.0358858.r005

**Authors:** Yury E Khudyakov

7 Sep 2026

The Role of Insulin Resistance in the Development of Hepatocellular Carcinoma with Computational Analysis of IRS-1 Interaction with HCV Genotype 3 Core Protein

PONE-D-26-01567R2

Dear Dr. Mujahid,

We’re pleased to inform you that your manuscript has been judged scientifically suitable for publication and will be formally accepted for publication once it meets all outstanding technical requirements.

Within one week, you’ll receive an e-mail detailing the required amendments. When these have been addressed, you’ll receive a formal acceptance letter and your manuscript will be scheduled for publication.

An invoice will be generated when your article is formally accepted. Please note, if your institution has a publishing partnership with PLOS and your article meets the relevant criteria, all or part of your publication costs will be covered. Please make sure your user information is up-to-date by logging into Editorial Manager at Editorial Manager® and clicking the ‘Update My Information' link at the top of the page. For questions related to billing, please contact billing support.

If your institution or institutions have a press office, please notify them about your upcoming paper to help maximize its impact. If they’ll be preparing press materials, please inform our press team as soon as possible -- no later than 48 hours after receiving the formal acceptance. Your manuscript will remain under strict press embargo until 2 pm Eastern Time on the date of publication. For more information, please contact onepress@plos.org.

Kind regards,

Yury E Khudyakov, PhD

Academic Editor

PLOS One

Additional Editor Comments (optional):

Reviewers' comments:

# Editor report: Acceptance letter

**DOI:** 10.1371/journal.pone.0358858.r006

**Authors:** Yury E Khudyakov

PONE-D-26-01567R2

PLOS One

Dear Dr. Mujahid,

I'm pleased to inform you that your manuscript has been deemed suitable for publication in PLOS One. Congratulations! Your manuscript is now being handed over to our production team.

At this stage, our production department will prepare your paper for publication. This includes ensuring the following:

* All references, tables, and figures are properly cited

* All relevant supporting information is included in the manuscript submission,

* There are no issues that prevent the paper from being properly typeset

You will receive further instructions from the production team, including instructions on how to review your proof when it is ready. Please keep in mind that we are working through a large volume of accepted articles, so please give us a few days to review your paper and let you know the next and final steps.

Lastly, if your institution or institutions have a press office, please let them know about your upcoming paper now to help maximize its impact. If they'll be preparing press materials, please inform our press team within the next 48 hours. Your manuscript will remain under strict press embargo until 2 pm Eastern Time on the date of publication. For more information, please contact onepress@plos.org.

You will receive an invoice from PLOS for your publication fee after your manuscript has reached the completed accept phase. If you receive an email requesting payment before acceptance or for any other service, this may be a phishing scheme. Learn how to identify phishing emails and protect your accounts at https://explore.plos.org/phishing.

If we can help with anything else, please email us at customercare@plos.org.

Thank you for submitting your work to PLOS One and supporting open access.

Kind regards,

PLOS One Editorial Office Staff

on behalf of

Dr. Yury E Khudyakov

Academic Editor

PLOS One
